# Mechanisms and Therapeutic Targets of Botanicals Derived from Asteraceae Plant Species

**DOI:** 10.3390/pharmaceutics18060759

**Published:** 2026-06-21

**Authors:** Aphelele Taliwe, Siphamandla Q. N. Lamula, Lisa V. Buwa-Komoreng, Vuyolwethu Khwaza

**Affiliations:** 1 Infectious Diseases and Medicinal Plants Research Niche Area, Botany Department, Faculty of Science and Agriculture, University of Fort Hare, Private Bag X1314, Alice 5700, South Africa; 202014249@ufh.ac.za (A.T.); slamula@ufh.ac.za (S.Q.N.L.); lbuwa@ufh.ac.za (L.V.B.-K.); 2 Chemistry Discipline, Department of Chemical and Earth Sciences, Faculty of Science and Agriculture, University of Fort Hare, Private Bag X1314, Alice 5700, South Africa

**Keywords:** Asteraceae, phytochemicals, molecular targets, anti-inflammatory, anticancer, molecular mechanisms, signaling pathways, antioxidants

## Abstract

The Asteraceae family represents one of the largest groups of medicinal plants, widely used in traditional medicine and increasingly investigated for its pharmacological potential. This review summarizes current evidence on the botanicals derived from Asteraceae plant species and their molecular mechanisms of action against inflammation and cancer. Major classes of bioactive compounds in extracts are discussed in relation to their modulation of key signaling pathways and therapeutic targets such as NF-κB, MAPK, PI3K/Akt, COX-2, iNOS, and apoptotic regulators (Bax/Bcl-2, caspases). A literature search covering studies published between 2022 and 2026 was conducted. Evidence from in vitro, in vivo, and in silico studies demonstrates that Asteraceae-derived botanicals exert therapeutic effects through antioxidant activity, cytokine suppression, enzyme inhibition, and regulation of gene expression. Overall, the mechanistic insights presented herein support the rational use of Asteraceae medicinal plants and identify promising lead compounds for drug discovery and development.

## 1. Introduction

Medicinal plants are a rich source of bioactive substances that have long been used for therapeutic purposes and as precursors for modern drug development. Historically, they have formed the backbone of traditional medical systems and continue to play a vital role in contemporary healthcare, particularly in developing regions where they are accessible, affordable, and widely perceived as safe and culturally acceptable therapeutic options [1,2,3]. In 2022, the World Health Organization (WHO) reported that 80% of the African population relies on plant-based remedies for primary healthcare needs [4]. The global herbal medicine market was valued at USD 148.5 billion in 2022 and is projected to grow from USD 165.13 billion in 2023 to USD 386.07 billion by 2032, with a compound annual growth rate of 11.20%. This growth is driven by increased demand following the COVID-19 pandemic, rising awareness of the side effects of allopathic medicines, growing preference for herbal alternatives, population expansion, and the increasing prevalence of chronic diseases [5]. Generally, plant-derived natural products are more environmentally friendly for frequent use when compared to synthesized drugs [6]. As a result, about 74% of FDA-approved drugs are either natural products or natural product-derived [7,8].

Among plant-based natural products, species of plants from the Asteraceae (daisy) family constitute one of the largest and most pharmacologically significant groups of angiosperms. It comprises over 43 tribes, approximately 1600 genera, and more than 33,000 species globally [9], including widely used medicinal plants such as chamomile (*Matricaria chamomilla*), feverfew (*Tanacetum parthenium*), and dandelion (*Taraxacum officinale*) [10,11,12]. Species within this family are characterized by a remarkable diversity of secondary metabolites such as flavonoids, terpenoids, phenolic acids, and sesquiterpene lactones, which have been associated with a broad spectrum of biological activities, including anti-inflammatory and anticancer effects [10,13]. Extensive research has been performed to study the therapeutic effect of extracts or phytochemicals derived from Asteraceae plant species to investigate their potential anticancer, anti-inflammatory and antioxidant activities. The outstanding results indicate that most Asteraceae plant species have potent anticancer [14,15], anti-inflammatory [16,17,18] and antioxidant [19,20] effects.

**Figure 1:**
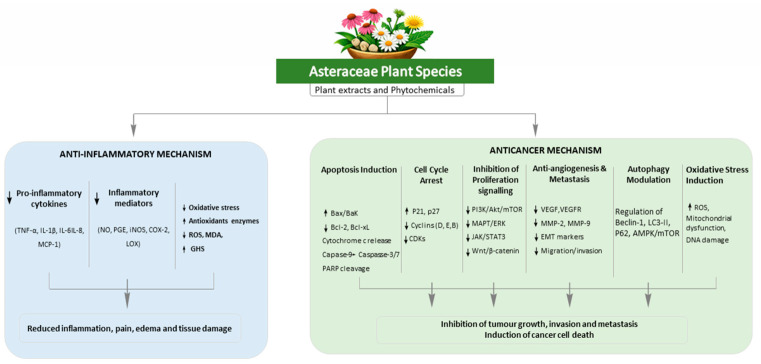
Schematic diagram showing different mechanisms and therapeutic targets of bioactive constituents derived from Asteraceae plant species. Note: “↑” indicates upregulation or increase; “↓” indicates downregulation or decrease.

Recent advances in molecular biology and pharmacological research have shifted attention from descriptive bioactivity studies toward elucidating the molecular mechanisms and therapeutic targets underlying the medicinal effects of Asteraceae-derived botanicals such as extracts and bioactive compounds. Emerging evidence indicates that these bioactive constituents modulate key signaling pathways, enzymes, transcription factors, and cellular processes involved in inflammation, cancer progression, and oxidative stress (Figure 1). However, despite growing interest, many Asteraceae species remain insufficiently studied at the mechanistic level, and gaps persist in understanding their target specificity, safety profiles, and translational potential [21].

Several recent reviews have explored the pharmacological potential of Asteraceae species, although their focus and scope differ from those of the present study. For instance, reviews by Rolnik and Olas (2021) [22] and Mohanta et al. [23] primarily provide broad overviews of the biological activities and phytochemical composition of selected Asteraceae species, with a strong focus on antioxidant and general therapeutic properties. Kazeminia et al. [24] and Awoke and Tahir [10] summarize ethnomedicinal uses and pharmacological activities but place comparatively less emphasis on the detailed molecular mechanisms and specific therapeutic targets underlying these effects. While these studies offer valuable insights into the pharmacological relevance of Asteraceae, they often remain descriptive in nature and do not systematically integrate mechanistic, target-based evidence across multiple disease pathways.

The present review adopts a mechanism-driven approach by critically analyzing how bioactive compounds and extracts from Asteraceae species modulate specific molecular targets, signaling pathways, and cellular processes involved in inflammation, cancer, and oxidative stress. This review highlights gaps in target validation, safety, and translational potential, thereby providing a more focused framework for future drug development. By bridging phytochemistry, pharmacology, and molecular targeting, this work aims to offer a more comprehensive and integrative perspective than prior reviews.

## 2. Methodology

### 2.1. Literature Search Strategy

A comprehensive literature search was conducted to identify published studies reporting on the pharmacological activities, molecular mechanisms, and therapeutic targets of botanicals derived from species belonging to the Asteraceae family. Four electronic databases, namely Google Scholar, Web of Science, PubMed, and Scopus, were searched for relevant literature published between January 2022 and March 2026. The final search was performed in March 2026.

The search strategy combined terms related to the Asteraceae family and its bioactive constituents with terms associated with pharmacological activities and molecular mechanisms. Keywords included “Asteraceae”, “Compositae”, “medicinal plants”, “phytochemicals”, “bioactive compounds”, “secondary metabolites”, “anti-inflammatory”, “anticancer”, “antioxidant”, “molecular targets”, “signaling pathways”, and “mechanisms of action”. Search terms were adapted where necessary to suit the indexing requirements of individual databases. Additional relevant publications were identified through manual examination of the reference lists of selected articles.

### 2.2. Study Selection Process

Articles were selected based on their relevance to the objectives of this review. Peer-reviewed original research articles and review papers published in English were considered. Studies investigating Asteraceae species, their extracts, isolated compounds, or phytochemical constituents with reported anti-inflammatory, anticancer, antioxidant, or related pharmacological activities were included. Particular emphasis was placed on studies that provided mechanistic insights into the modulation of molecular targets, signaling pathways, enzymes, transcription factors, cytokines, or other biological processes associated with disease prevention or treatment.

Publications not written in English, conference abstracts, editorials, book reviews, and studies lacking sufficient experimental detail were excluded. Studies not directly related to the pharmacological activities or molecular mechanisms of Asteraceae-derived botanicals were also omitted.

### 2.3. Data Extraction and Synthesis

Relevant information was extracted from the selected literature, including plant species, plant parts used, type of extract or isolated compound, experimental model, biological activity, molecular targets, signaling pathways, and principal findings. The collected data were organized according to therapeutic activity and mechanistic pathways to facilitate comparison across studies.

The findings were synthesized narratively, with particular emphasis on identifying common molecular mechanisms and therapeutic targets associated with Asteraceae-derived botanicals. The review further discusses the relationships between phytochemical composition and biological activity, highlighting emerging trends, research gaps, and opportunities for future drug discovery and development.

### 2.4. Critical Evaluation of the Literature

Although this review does not constitute a systematic review, the quality and scientific relevance of the included studies were critically considered during data interpretation. Factors such as experimental design, methodological rigor, reproducibility of findings, appropriateness of biological models, and the strength of mechanistic evidence were taken into account. Greater emphasis was placed on studies supported by multiple experimental approaches, including in vitro, in vivo, and in silico investigations. Potential limitations, including insufficient mechanistic validation, limited toxicity evaluation, and the lack of clinical evidence, were also considered when drawing conclusions and identifying future research directions.

## 3. Botanical and Ethnopharmacological Background of the Asteraceae Family

The Asteraceae (Compositae) family is one of the largest and most diverse plant families, comprising approximately 33,000 recognized species distributed across 1600 genera worldwide, with the exception of the Antarctic [9,21]. Often described as cosmopolitan, this family is well adapted to a broad range of ecological zones, including temperate, cold-temperate, subtropical, and high-altitude regions, though species are relatively less common in tropical environments [25]. Members of the Asteraceae family thrive from sea level to mountainous terrains and are easily distinguished by their characteristic inflorescence, a capitulum composed of multiple compact florets.

The morphology of Asteraceae plants is highly diverse. While most species are perennial or annual herbs, the family also includes shrubs and trees that may exceed 30 m in height [11,22]. Leaf structure varies considerably: some species possess large foliage, others are small and spiny, and a few are nearly leafless, relying on photosynthetic stems instead. Leaves typically bear trichomes or glandular hairs of varying lengths and pigmentation [11,22,25].

Economically, Asteraceae ranks among the most significant plant families. Numerous species serve as food crops (*Lactuca sativa*—lettuce), herbal teas (*Chamomilla recutita*—chamomile), ornamentals, and commercial sources of oils, dyes, insecticides, and cosmetics [10]. Many species have been widely used in traditional medicine and as food sources across diverse cultures for centuries [22,26]. The family also contributes to human nutrition, providing 0.4–6.13 g of protein and 2.55–13.44 g of dietary fiber per 100 g of edible material, alongside Na, K, Ca, Mg, and vitamins A–D. Most species are naturally low in fat [11,21,23,24].

Owing to their extensive ethnomedicinal relevance and diverse phytochemical composition, Asteraceae plants continue to attract scientific attention for the management of infections, inflammation, oxidative stress-related disorders, metabolic diseases, and other pathological conditions. A summary of notable medicinal species and their traditional uses is presented in Table 1.

## 4. Phytochemical Constituents of Plants in the Asteraceae Family

This section discusses the diverse phytochemical constituents of Asteraceae medicinal plants that contribute to their pharmacological activities. More than 5000 bioactive compounds have been identified within this family, many of which exhibit notable pharmacological properties [65]. These constituents include secondary metabolites, essential oils, volatile compounds, and a wide array of structural classes such as sesquiterpene lactones, diterpenes, triterpenes, inulin-type fructans, polyacetylenes, pentacyclic triterpene alcohols, benzofurans, flavonoids, flavones, and unsaturated fatty acids [66,67].

Asteraceae species are particularly rich in phenolic acid derivatives, terpenoids, flavonoids, sesquiterpene lactones, and polyacetylenes. Phenolic acids and flavonoids are powerful antioxidants known for antibacterial, anti-inflammatory, anticancer, and enzyme inhibitory activities, while sesquiterpene lactones possess strong anti-inflammatory and anticancer effects, though some can induce allergic dermatitis [22]. Commonly reported secondary metabolites include macrocephalides, lupeol, α-amyrin, β-amyrin, pseudotaraxasterol, and multiple caffeic acid derivatives associated with broad biological activity.

Other phytochemicals of significance include coumarins, polyphenols, fructooligosaccharides, vitamins, and highly specialized compounds such as artemisinin from *Artemisia annua*, steviol glycosides from *Stevia rebaudiana*, helenalin from *Arnica montana*, parthenolide from feverfew, and silymarin from *Silybum marianum* [68]. *Taraxacum officinale* and *T. mongolicum*, for instance, are rich in triterpenoids such as α-/β-amyrin, taraxasterol, arnidiol, ψ-taraxasterol, cardiol, and taraxacinic acid, along with sterols like β-sitosterol and stigmasterol, compounds linked to anti-inflammatory, antidiabetic, and anticancer activities [69,70]. These species further contain organic acids including malic, citric, fumaric, oxalic, ascorbic, and quinic acids, enhancing their therapeutic value.

*Arctium lappa* is notable for arctigenin and arctiin, lignans widely investigated for their ability to modulate inflammatory pathways. Similarly, *Bidens bipinnata* exhibits a chemically rich profile, containing large quantities of tannins, flavonoids, saponins, phenols, alkaloids, lipids, oxalates, and cyanogenic glycosides, supporting its antioxidant, anti-inflammatory, analgesic, immunomodulatory, and anticancer properties [71].

*Matricaria pubescens* demonstrates a broad secondary metabolic spectrum including essential oils (EOs), phenolic acids, flavonoids, coumarins, and saponins. GC–MS profiling of aerial parts has identified volatile monoterpenes and sesquiterpenes such as β-ocimene, α-pinene, spathulenol, and limonene, along with notable flavonoid aglycones including apigenin, luteolin, and quercetin, which contribute significantly to its antioxidant and antimicrobial activities [57].

*Asteriscus graveolens* is rich in flavonoids (myricetin, apigenin derivatives, luteolin glycosides, rutin, hyperoside) and EO constituents such as kessane, myrtenyl acetate, cis-chrysanthenyl acetate, carvacrol, camphor, α-thujone, and p-cineole, supporting its broad pharmacological potential including antibacterial, anticancer, antihypertensive, antidiabetic, antileishmanial, and antioxidant effects [72,73]. *Echinops kebericho* is particularly valued for dehydrocostus lactone, a potent antibacterial and antioxidant sesquiterpene lactone. Conversely, *Matricaria chamomilla* contains significant levels of α-bisabolol and chamazulene, which are beneficial in treating oral and inflammatory conditions such as gingivitis and stomatitis [74].

*Silybum marianum* consists predominantly of the silymarin complex, including silibinin, isosilybin A/B, silychristin, silydianin, and taxifolin, along with flavonoid glycosides [75]. The plant also provides an essential fatty acid profile dominated by linoleic and oleic acids, with additional palmitic and behenic acids. In *Baccharis discolor*, diverse classes including alkaloids, phenolic acids, flavonoids, steroids, saponins, sesquiterpene lactones, terpenoids, and triterpenoids such as α-amyrin acetate, β-amyrin acetate, lupeol, and taraxasterol have been reported, supporting antioxidant and anti-inflammatory applications [61].

*Tussilago farfara* contains more than 184 structurally diverse phytochemicals, including fatty acids, carbohydrates (inulin, sucrose, and raffinose), pectins, latex, phytosterols (sitosterol and stigmasterol), alkaloids, phenolic acids, sesquiterpenoids (tussilagone and hydroxytussilane) and flavonoids (quercetin, rutin, hyperoside, and kaempferol). *Echinops kebericho* further displays three defined chemotypes, varying in their dominant volatile constituents, all driven by the biologically essential α-methylene-γ-lactone moiety in dehydrocostus lactone [74].

## 5. Molecular Mechanisms and Therapeutic Targets of Asteraceae Botanicals

Asteraceae botanicals such as plant extracts or bioactive compounds/phytochemicals exert their therapeutic effects by modulating multiple signaling pathways and molecular targets such as in inflammation, cancer progression, oxidative stress, metabolic dysfunction, and microbial pathogenesis. These mechanisms are largely mediated by secondary metabolites such as sesquiterpene lactones, flavonoids, terpenoids, and phenolic acids, which interact with different enzymes, intracellular signaling cascades and transcription factors [76]. This review article focused on summarizing recent research studies published in English from the last five years (2022–2026) focusing on the molecular mechanisms and therapeutic targets of botanicals derived from Asteraceae medicinal plants, highlighting their potential anticancer and anti-inflammatory activities.

### 5.1. Anti-Inflammatory Mechanisms/Targets of Plant Extracts

Inflammation is a complex physiological process mediated by numerous biochemical factors, including pro-inflammatory enzymes such as cyclooxygenase (COX) and lipoxygenase (LOX), cytokines like TNF-α and IL-1β, and central transcription factors such as NF-κB [77]. Clinically, non-steroidal anti-inflammatory drugs are commonly used to treat inflammation, but their adverse effects, such as gastrointestinal irritation, platelet dysfunction, and nephrotoxicity, become potential threats to patients’ health [78]. Hence, there is an urgent need to develop anti-inflammatory drugs that are both safe and effective. Natural products have emerged as a promising source for discovering new drug candidates due to their effectiveness and safety profile. Increasing evidence highlights the significant potential of natural medicines in the treatment of diseases linked to inflammation [79]. Plant-derived compounds capable of modulating pathways have become increasingly important in the search for new therapeutic agents. Members of the Asteraceae family, in particular, demonstrate notable anti-inflammatory activities through diverse and often complementary mechanisms of action. Numerous Asteraceae botanicals, particularly extracts and their bioactive compounds suppress NF-κB activation by inhibiting IκB kinase phosphorylation and preventing nuclear translocation of NF-κB subunits [80,81]. This leads to reduced expression of pro-inflammatory mediators including TNF-α, IL-1β, IL-6, COX-2, and iNOS [82,83].

#### 5.1.1. NF-κB and MAPK Signaling Pathways

Several studies have demonstrated that plant extracts suppress inflammatory mediators through modulation of NF-κB and MAPK pathways. Ngabire et al. [84] reported that the methanolic extract of *Kalimeris incisa* significantly reduced NO production, iNOS, COX-2, TNF-α, IL-1β, and IL-6 levels in LPS-stimulated RAW 264.7 macrophages. Mechanistically, the extract inhibited phosphorylation of PI3K, IκBα, p65 NF-κB, ERK1/2, SAPK/JNK, and Akt, indicating simultaneous modulation of NF-κB, MAPK, and Akt pathways. Similarly, Kang et al. [85] demonstrated that the aqueous extract of *Ixeridium dentatum* (Thunb.) Tzvelev (Asteraceae) suppressed TNF-α, COX-2, iNOS, IL-6, NF-κB, and IL-1β expression in activated macrophages, confirming its anti-inflammatory potential through regulation of inflammatory signaling mediators. Song et al. [86]. further showed that FLEXA, an herbal formulation containing *Carthamus tinctorius*, inhibited both MAPK and NF-κB pathways in SW1353 osteoarthritis cells, leading to decreased cytokine production and extracellular matrix degradation.

**Figure 2:**
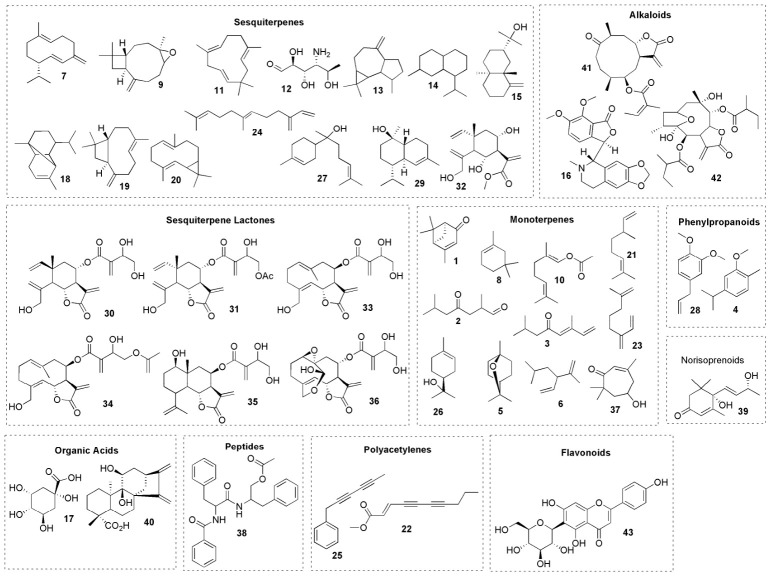
Chemical structures of bioactive compounds identified from the reported Asteraceae plant species with potential anti-inflammatory activity (compounds **1**–**43**).

González-Velasco et al. [87] identified verbenone (**1**), dihydrotagetone (**2**), and tagetone (**3**) (Figure 2) as the major constituents of *Tagetes parryi* EO. Both the EO and compound **1** significantly reduced TPA-induced ear edema and inhibited NO, TNF-α, and IL-6 production in LPS-stimulated macrophages. The antinociceptive effects observed in writhing and formalin assays were reversed by L-NAME, suggesting involvement of nitric oxide signaling. Similarly, Do et al. [88] demonstrated that EOs from *Blumea lanceolaria* inhibited NO production and suppressed IL-6, TNF-α, COX-2, and iNOS expression in RAW 264.7 macrophages. Carvacrol methyl ether (**4**, Figure 2) was identified as the major active compound in both leaves and stems. Mechanistic analysis revealed inhibition of NF-κB activation through suppression of IκBα and p65 phosphorylation, while molecular docking suggested interactions with NF-κB, MAPK, and arachidonic acid metabolism pathways. Lin et al. [89] also reported that EO from the leaves of *Crossostephium chinense* (Makino) L. (Asteraceae) reduced NO, ROS, TNF-α, IL-6, and IL-1β production through modulation of ERK/MAPK and NF-κB signaling pathways. Three bioactive compounds such as 1,8-cineole (**5**), santolina triene (**6**), and germacrene D (**7**) (Figure 2) were identified from *C. chinense* EO.

#### 5.1.2. Antioxidant and Cytoprotective Pathways

Activation of antioxidant and cytoprotective pathways has also been linked to the anti-inflammatory effects of several extracts. Lin et al. [89] showed that *C. chinense* EO activated the NRF2/HO-1 pathway while suppressing inflammatory mediators, suggesting dual antioxidant and anti-inflammatory effects. Ticolea et al. [90] demonstrated that the ethanolic extract from the aerial parts of *Artemisia alba Turra* reduced oxidative stress markers and downregulated NLRP3 inflammasome-associated proteins, including gasdermin D, caspase-1, NF-κB-p65, IL-18, and IL-1β. The extract also displayed antiproliferative activity against ovarian cancer cells and protected against inflammation-associated renal injury without hepatotoxicity. Kawada et al. [91] further demonstrated that exosome-like nanoparticles isolated from the rhizome of *Atractylodes lancea* suppressed NO and pro-inflammatory cytokine production while enhancing expression of protective genes such as heme oxygenase-1 in microglial cells, highlighting their neuroprotective anti-inflammatory potential.

#### 5.1.3. Enzymatic Targets (COX-2, iNOS, and 5-LOX)

Several studies also highlighted inhibition of COX and LOX enzymes as important anti-inflammatory mechanisms. Göç et al. [92] reported that ethyl acetate extracts of *Scorzonera ketzkhowelii* strongly inhibited COX-I and COX-II activity, with effects comparable to indomethacin, supporting previous reports on the anti-inflammatory properties of *Scorzonera* species [93,94]. Younis et al. [95] demonstrated that aqueous and dichloromethane extracts of *Cynara cardunculus* exhibited potent inhibitory effects against COX-2 and 5-LOX enzymes, suggesting suppression of arachidonic acid metabolism pathways. Das et al. [96] also showed that compounds such as α-pinene (**8**), caryophyllene oxide (**9**), and geranyl acetate (**10**) (Figure 2) from *Tagetes erecta* interacted strongly with inflammatory targets including iNOS, TNF-α, and IL-6 in silico (molecular docking studies).

#### 5.1.4. Cytokine Modulation and Immune Response

Anti-inflammatory effects associated with cytokine modulation and inhibition of immune cell migration were also observed. Sánchez-Canul et al. [97] demonstrated that the methanolic extract of *Montanoa grandiflora*, rich in terpenes such as β-caryophyllene (**11**), (-)-α-cubebene (**12**), alloaromadendrene (**13**), (+)-δ-cadinene (**14**) and β-eudesmol (**15**), along with the alkaloid (±)-nor-β-hydrastine (**16**), quinic acid (**17**) (Figure 2), carbohydrates, and fatty acids, increased IL-10 levels while reducing TNF-α, IL-1β, IL-6, NO, and H_2_O_2_ production. The extract also inhibited carrageenan-induced paw edema, TPA-induced ear edema, and delayed-type hypersensitivity reactions without cytotoxic or hemolytic effects. Likewise, Machado et al. [98] demonstrated that EO from *Vernonanthura tweediana*, containing sesquiterpenes such as α-copaene (**18**), β-caryophyllene (**11**), α-caryophyllene (**19**), germacrene D (**7**), and bicyclogermacrene (**20**) (Figure 2) reduced leukocyte migration and regulated cytokine secretion, particularly TNF-α, IL-1β, and IL-6. These activities were mainly attributed to the high concentration of caryophyllene. Tawfik et al. [99] similarly reported that EOs from *Achillea fragrantissima* suppressed IL-2, TNF-α, IL-6, and iNOS expression in activated macrophages, suggesting potential applications in inflammatory skin disorders.

#### 5.1.5. Multi-Target and Synergistic Mechanisms

Several studies combined anti-inflammatory activity with cytotoxic or antiproliferative effects. An et al. [100] reported that EOs from *Erigeron sublyratus* (Roxb. Ex) DC. (Asteraceae), containing β-caryophyllene (**11**), germacrene D (**7**), trans-β-ocimene (**21**), cis-lachnophyllum ester (**22**), β-myrcene (**23**) and (E)-β-farnesene (**24**) (Figure 2), significantly reduced NO production in macrophages and exhibited cytotoxicity against HepG2, MCF-7, and SK-LU-1 cancer cell lines. Molecular docking revealed strong interactions between compound **24** and VEGFR-2, and between compound **23** and COX-2. Zhang et al. [101] further demonstrated that EO from *Artemisia scoparia*, dominated by capillen (**25**, Figure 2), inhibited MPO, NO, and TNF-α production more effectively than dexamethasone at lower concentrations. Network pharmacology and docking studies identified L-α-terpineol (**26**), α-bisabolol (**27**), methyleugenol (**28**), and α-cadinol (**29**) (Figure 2) as major bioactive constituents interacting with PPARA, PPARG, PTGS2, ESR1, EP300, and HMGCR, indicating involvement of cAMP, PPAR, serotonergic synapse, and neuroactive ligand–receptor interaction pathways. Ali et al. [102] also reported that EO from *Artemisia abrotanum* significantly inhibited NO production and iNOS expression in a concentration-dependent manner, further supporting the anti-inflammatory potential of Asteraceae-derived EOs.

Evidence gathered from the reviewed studies suggests that the anti-inflammatory effects of Asteraceae-derived extracts arise from the modulation of several complementary biological pathways, as summarized in Table 2. Although the specific mechanisms varied among species and extract types, inhibition of NF-κB and MAPK signaling was consistently associated with reduced production of inflammatory mediators, including NO, TNF-α, IL-1β, IL-6, COX-2, and iNOS. Many extracts also enhanced cellular antioxidant defenses through activation of the Nrf2/HO-1 pathway, highlighting the close relationship between oxidative stress regulation and inflammation control. In addition, suppression of COX and LOX enzyme activity indicates that these botanical preparations can influence arachidonic acid metabolism and limit the formation of pro-inflammatory mediators. Taken together, the findings point to a multifaceted mode of action in which several molecular targets contribute to the overall anti-inflammatory response.

The studies reviewed also highlight the considerable chemical diversity present within the Asteraceae family. A wide range of bioactive constituents, including sesquiterpenes, monoterpenes, phenolic compounds, alkaloids, and other secondary metabolites, have been implicated in anti-inflammatory activity. Among these, compounds such as β-caryophyllene, germacrene D, 1,8-cineole, carvacrol methyl ether, and α-bisabolol were frequently identified as contributors to the observed biological effects. Their repeated occurrence across different species and experimental models suggests that they warrant further investigation as potential candidates for drug discovery. Notably, several studies reported overlapping antioxidant, anti-inflammatory, antiproliferative, and immunomodulatory activities, indicating that the therapeutic potential of Asteraceae species may extend beyond the regulation of inflammation alone. Such multifunctional properties could be particularly relevant in complex disorders where chronic inflammation contributes to disease progression, including cancer and neuroinflammatory conditions.

### 5.2. Anti-Inflammatory Mechanisms/Targets of Phytochemicals

Bioactive phytochemicals isolated from Asteraceae species also exert anti-inflammatory effects through modulation of inflammatory enzymes, cytokines, oxidative stress pathways, and transcription factors.

#### 5.2.1. Lipoxygenase (LOX) Inhibition and Suppression of Arachidonic Acid Metabolism

Several phytochemicals isolated from Asteraceae species demonstrated anti-inflammatory activity through inhibition of LOX, a key enzyme involved in arachidonic acid metabolism and inflammatory mediator production.

Dimitriadis et al. [103] isolated several sesquiterpene lactones, germacranolides, and eudesmanolides from *Centaurea bruguieriana*. Among the isolated metabolites, 8α-O-(3,4-dihydroxy-2-methylenebutanoyloxy) dehydromelitensine (**30**), 8α-O-(3-hydroxy-4-acetoxy-2-methylene-butanoyloxy) dehydromelitensine (**31**), methyl 6α,8α,15-trihydroxyelema-1,3,11(13)-trien-12-oate (**32**), cnicin (**33**), 4′-O-acetylcnicin (**34**), and malacitanolide (**35**) exhibited strong LOX inhibitory activity, with docking scores exceeding those of nordihydroguaiaretic acid. These findings suggest potent anti-inflammatory activity mediated through suppression of arachidonic acid metabolism.

#### 5.2.2. Cytokine Modulation

Regulation of pro- and anti-inflammatory cytokines was another important anti-inflammatory mechanism.

Yazdani et al. [104] isolated several compounds from the methanolic extract of *Vernoniastrum migeodii* and evaluated their effects in LPS-stimulated A549 cells. Among the isolated metabolites, 3′-hydroxylvernolide (**36**), eucarvone (**37**), and aurantiamide acetate (**38**) significantly reduced IL-6 mRNA expression, indicating anti-inflammatory activity through suppression of pro-inflammatory cytokine signaling. However, (6S,9R)-vomifoliol (**39**) increased IL-6 and IL-8 levels, demonstrating differential modulation of inflammatory pathways. Adenostemmoic acid B (**40**), isolated from *Gymnocoronis spilanthoides*, reduced TNF secretion and increased IL-10 levels in activated macrophages, further supporting its immunomodulatory and anti-inflammatory properties.

Abdel-Baki et al. [105] demonstrated that treatment with extracts and isolated compounds from *Achillea santolina* reduced expression of inflammatory cytokines including IL-1β and IL-10, contributing to both anti-inflammatory and neuroprotective effects.

#### 5.2.3. NF-κB Signaling Inhibition

Inhibition of NF-κB signaling emerged as a central anti-inflammatory mechanism. Adenostemmoic acid B (**40**, Figure 2) suppressed inflammatory responses through inhibition of NF-κB and IRF transcription factors in activated macrophages [106]. Combination treatment with benznidazole further enhanced inhibition of NF-κB signaling and nitrite production [106]. Carpelipine C (**41**, Figure 2) and carpelipine D (**42**, Figure 2), isolated from *Carpesium lipskyi*, also exerted anti-inflammatory effects through modulation of NF-κB signaling pathways in LPS-stimulated RAW264.7 macrophages [107].

#### 5.2.4. MAPK Pathway Modulation

Carpelipine C (**41**) and carpelipine D (**42**) additionally modulated MAPK signaling pathways, contributing to suppression of inflammatory responses in RAW264.7 cells [107].

#### 5.2.5. Suppression of Nitric Oxide (NO) Production and iNOS Expression

Several phytochemicals reduced inflammatory NO production by suppressing inducible nitric oxide synthase (iNOS). Adenostemmoic acid B (**40**) significantly reduced NO production in activated macrophages [106]. Similarly, carpelipine C (**41**) and carpelipine D (**42**) inhibited NO production and suppressed iNOS expression in LPS-stimulated RAW264.7 cells [107].

#### 5.2.6. COX-2 Inhibition

COX-2 inhibition was another important anti-inflammatory target. Carpelipine C (**41**) and carpelipine D (**42**) suppressed COX-2 expression in activated macrophages [107]. Abdel-Baki et al. [105] further demonstrated that extracts and compounds from *Achillea santolina* reduced COX-2 gene expression.

Molecular docking studies showed that isovitexin (**43**, Figure 2) exhibited strong binding affinity toward COX-2 and 5-LOX, supporting its multitarget anti-inflammatory activity [105].

#### 5.2.7. Activation of Nrf2/HO-1 Antioxidant Pathway

Activation of antioxidant defense pathways contributed to the anti-inflammatory effects of some phytochemicals. Carpelipine C (**41**) and carpelipine D (**42**) activated the Nrf2/HO-1 antioxidant pathway, which was associated with suppression of inflammatory signaling and oxidative stress [107]. Similarly, molecular docking analysis revealed that isovitexin (**43**) strongly interacted with HO-1 and Keap1, suggesting regulation of the Nrf2/HO-1 antioxidant system [105].

#### 5.2.8. Multitarget Anti-Inflammatory and Neuroprotective Effects

Isovitexin (**43**) isolated from *Achillea santolina* exhibited multitarget activity through interactions with HO-1, 5-LOX, Keap1, and COX-2, supporting both anti-inflammatory and neuroprotective effects [105]. Treatment also alleviated glutamate-induced neurotoxicity, indicating combined antioxidant and anti-inflammatory neuroprotection [105].

#### 5.2.9. IRF Transcription Factor Inhibition

Adenostemmoic acid B (**40**) inhibited interferon regulatory factor (IRF) transcription signaling in activated macrophages, contributing to suppression of inflammatory responses [106].

#### 5.2.10. Combination Therapy and Enhanced Anti-Inflammatory Activity

Combination treatment of adenostemmoic acid B (**40**) with benznidazole enhanced suppression of NF-κB signaling and nitrite production, suggesting synergistic anti-inflammatory activity and potential utility in inflammatory disease management [106].

The evidence summarized in Table 3 indicates that phytochemicals isolated from Asteraceae species modulate inflammatory responses through multiple, often overlapping, molecular pathways. Among the most frequently reported mechanisms was the regulation of NF-κB and MAPK signaling, which was associated with reduced production of inflammatory mediators such as NO, TNF-α, IL-6, iNOS, and COX-2. Several compounds also displayed inhibitory activity against LOX and COX enzymes, highlighting the importance of arachidonic acid metabolism as a target for anti-inflammatory intervention. In addition to experimental findings, molecular docking studies identified compounds including cnicin, malacitanolide, and isovitexin as having favorable interactions with key inflammatory targets.

The available evidence reflects the remarkable structural diversity of anti-inflammatory constituents identified within the Asteraceae family, encompassing sesquiterpene lactones, germacranolides, flavonoids, and various terpenoid-derived compounds. Despite sharing anti-inflammatory potential, these metabolites appear to act through different biological mechanisms. Some primarily influence the production of inflammatory cytokines, whereas others are more closely associated with the regulation of antioxidant defenses and cytoprotective pathways, including Nrf2/HO-1 signaling. Not all compounds, however, produced favorable outcomes. For example, (6S,9R)-vomifoliol was reported to increase IL-6 and IL-8 expression, suggesting a pro-inflammatory effect under the tested conditions [104]. Such findings emphasize the complexity of phytochemical activity and demonstrate the need for careful mechanistic characterization rather than assuming uniform therapeutic benefit across structurally related compounds. Therefore, comprehensive pharmacological assessment, including safety and toxicity profiling, remains an essential step in the identification and development of promising lead molecules.

We have noted that the reviewed studies also demontrated the ability of several isolated phytochemicals to influence multiple biological processes simultaneously. Beyond their anti-inflammatory effects, a number of compounds also exhibited antioxidant, neuroprotective, and immunomodulatory properties, reflecting the complex pharmacological profiles often associated with plant-derived metabolites. A good example is isovitexin, which was reported to interact with targets including HO-1, Keap1, 5-LOX, and COX-2, indicating potential involvement in both oxidative stress regulation and inflammatory signaling [105]. Adenostemmoic acid B also showed enhanced activity when administered in combination with benznidazole, suggesting a possible synergistic interaction [106]. The capacity of these compounds to act on several interconnected pathways may be particularly relevant in chronic inflammatory conditions, where oxidative stress, immune imbalance, and progressive tissue injury frequently contribute to disease development and persistence.

Although the current findings are encouraging, much of the available evidence originates from cell-based experiments, molecular docking analyses, and early-stage pharmacological investigations. As a result, important questions regarding pharmacokinetic behavior, bioavailability, long-term safety, and therapeutic effectiveness under physiological conditions remain unresolved. Future studies should place greater emphasis on in vivo validation, toxicity assessment, and structure–activity relationship analyses to clarify the translational potential of these compounds. Addressing these gaps will be essential for determining whether the observed biological activities can be reproduced in clinical settings. Even with these limitations, the body of evidence accumulated to date highlights Asteraceae-derived phytochemicals as a promising reservoir of bioactive molecules that could contribute to the development of new anti-inflammatory therapeutics.

When comparing the evidence presented in Section 5.1 and Section 5.2, it is evident that both crude extracts and isolated phytochemicals derived from Asteraceae species possess important anti-inflammatory properties, even though their modes of action and pharmacological implications differ. Studies involving crude extracts and EO generally reflected the combined effects of multiple constituents acting simaltaneously. Therefore, these preparations often influenced several biological pathways simultaneously, including NF-κB, MAPK, Nrf2/HO-1, COX, and LOX signaling, while also demonstrating antioxidant, immunomodulatory, and cytoprotective activities. Such findings suggest that phytochemical synergy may play an important role in the overall therapeutic response.

The investigation of isolated compounds provided a more precise understanding of the molecular mechanisms responsible for anti-inflammatory activity. The characterization of individual metabolites, including sesquiterpene lactones, germacranolides, flavonoids, etc., enabled the direct evaluation of their interactions with specific enzymes, transcription factors, and inflammatory mediators. This approach offers valuable insight into structure–activity relationships and facilitates the identification of compounds with potential for further pharmacological development. Together, these complementary lines of evidence highlight the importance of studying both complex botanical preparations and purified phytochemicals when exploring the therapeutic potential of Asteraceae species.

### 5.3. Structure–Activity Relationships of Major Phytochemical Classes with Anti-Inflammatory Properties

#### 5.3.1. Monoterpenes (C_10_ Compounds)

Monoterpenes such as verbenone (**1**), dihydrotagetone (**2**), tagetone (**3**), and 1,8-cineole (**5**) are relatively small and lipophilic, allowing efficient membrane permeability and rapid interaction with intracellular targets [87]. Oxygenated monoterpenes (e.g., verbenone, terpinenols, and cineole) generally exhibit stronger anti-inflammatory activity than hydrocarbon monoterpenes (e.g., β-myrcene and trans-β-ocimene). This is attributed to the presence of functional groups such as ketones, alcohols, and epoxides, which enhance binding to enzymes like COX-2 and iNOS [108,109]. Their mechanisms often involve inhibition of NO production and suppression of NF-κB and MAPK signaling pathways [108,109]. In contrast, non-oxygenated monoterpenes mainly contribute through weaker hydrophobic interactions.

#### 5.3.2. Sesquiterpenes (C_15_ Compounds)

Sesquiterpenes, including β-caryophyllene (**11**), germacrene D (**7**), α-bisabolol (**27**), and α-cadinol (**29**), show enhanced biological activity compared to monoterpenes due to their larger size and increased structural complexity. Hydrocarbon sesquiterpenes (e.g., caryophyllene, and farnesene) primarily act through hydrophobic interactions with membrane-associated receptors and enzymes, whereas oxygenated sesquiterpenes (e.g., eudesmol (**15**), bisabolol, cadinol) demonstrate stronger activity due to the presence of hydroxyl groups that facilitate hydrogen bonding with protein targets [110,111]. Notably, β-caryophyllene acts as a CB2 receptor agonist, contributing to anti-inflammatory and immunomodulatory effects. Overall, increased lipophilicity and functionalization enhance their ability to modulate cytokine production and inhibit inflammatory mediators [112].

#### 5.3.3. Phenylpropanoids/Aromatic Compounds

Phenylpropanoids such as methyleugenol (**28**) and carvacrol methyl ether (**4**) exhibit strong anti-inflammatory activity due to their aromatic ring systems combined with methoxy or phenolic substituents [88,101]. These structural features enable resonance stabilization and effective radical scavenging, contributing to antioxidant and anti-inflammatory effects. Their lipophilic aromatic framework also facilitates interaction with transcription factors such as NF-κB and enzymes like COX-2, resulting in suppression of pro-inflammatory cytokines [113,114,115].

#### 5.3.4. Alkaloids

Alkaloids such as (±)-nor-β-hydrastine (**16**) and carpelipine C (**41**) contain nitrogen atoms that enhance their ability to interact with biological macromolecules through ionic and hydrogen bonding interactions. These compounds often show strong enzyme inhibition (e.g., iNOS, COX-2) and modulation of signaling pathways such as NF-κB and MAPK. Their structural rigidity and heterocyclic frameworks contribute to selective binding and potent anti-inflammatory effects [107,116,117].

#### 5.3.5. Organic Acids and Related Compounds

Compounds such as quinic acid (**17**) and adenostemmoic acid B (**40**) are more polar and often exhibit indirect anti-inflammatory effects through antioxidant mechanisms. Their multiple hydroxyl and carboxyl groups enhance free radical scavenging and modulation of oxidative stress-related pathways such as Nrf2/HO-1 [118]. However, their lower lipophilicity may limit membrane permeability compared to terpenoids [119].

#### 5.3.6. Polyacetylenes

Polyacetylenes such as capillen (**25**) and cis-lachnophyllum ester (**22**) are characterized by conjugated triple bonds, which confer high chemical reactivity. These structural features allow interaction with nucleophilic sites in proteins, potentially modulating inflammatory enzymes and signaling pathways [120,121]. Their activity is often linked to electrophilic properties and the ability to disrupt cellular redox balance.

### 5.4. Anticancer Mechanisms/Targets of Asteraceae Plant Extracts

Cancer is characterized by the uncontrolled proliferation of abnormal cells that can invade surrounding tissues and metastasize to distant organs. According to the International Agency for Research on Cancer, in 2012, there were 14.1 million new cancer cases, 8.2 million cancer-related deaths, and 32.6 million people living with the disease worldwide. Projections indicated that by 2030, new cancer cases could reach 26 million, with approximately 17 million deaths per year [122]. The standard approach to treating cancer typically involves a combination of chemotherapy, surgery, radiotherapy, and, in other cases, hormone therapy. However, these strategies face several challenges, such as non-specific targeting of cells, the development of multidrug resistance, low absorption, cancer recurrence, and generally poor treatment outcomes [123]. As a result, there is a continuing need to develop novel anticancer agents that are both effective and affordable. Natural products have long been explored as a valuable source of potential anticancer agents. Many studies have validated the anticancer efficacy of natural plant products.

Numerous studies have investigated the anticancer properties of botanicals derived from Asteraceae species. Various studies have demonstrated that extracts and phytochemicals derived from Asteraceae species can modulate the cellular transcription factors, growth factor receptors, inflammatory cytokines, and many other molecular targets involved in regulating the cell proliferation, metastasis, apoptosis, angiogenesis, and autophagy through different signaling pathways [124,125,126,127,128,129]. These molecular events result in programmed cell death and suppression of tumor progression. The reviewed studies investigated various anticancer mechanisms or therapeutic targets associated with plant extracts and isolated phytochemicals from Asteraceae species.

#### 5.4.1. Apoptosis Induction by Plant Extracts 

**Figure 3:**
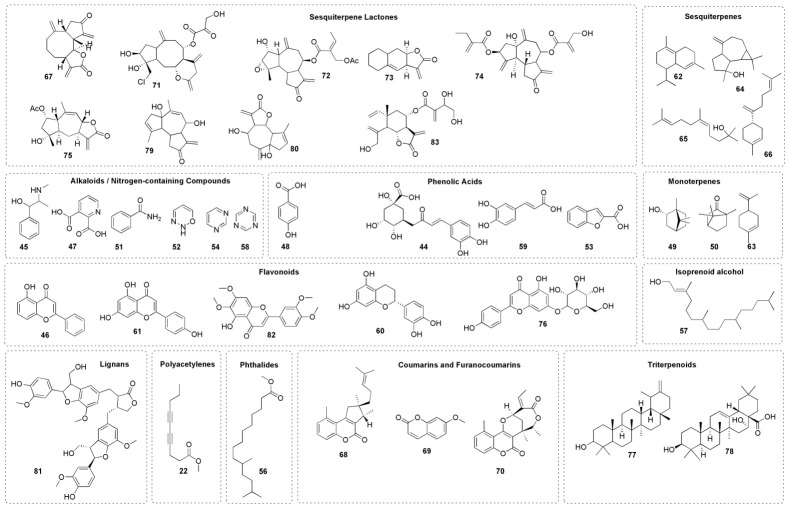
Chemical structures of identified bioactive compounds from Asteraceae medicinal plants with potential anticancer activity (compounds **44**–**83**).

Induction of apoptosis is one of the most consistently reported anticancer mechanisms of Asteraceae-derived extracts and phytochemicals. Across multiple studies, both intrinsic (mitochondrial-mediated) and extrinsic apoptotic pathways were activated through modulation of Bax, Bcl-2, cytochrome c, caspases, and mitochondrial membrane potential. For example, extracts of *Achillea multifida* induced apoptosis, DNA damage and mitotic catastrophe in MCF-7 and MDA-MB-231 cells, with chlorogenic acid (**44**, Figure 3) identified as a major active constituent [130]. Silver chloride nanoparticles synthesized using *Artemisia sieberi* extract promoted apoptosis in MDA-MB-231 cells through increased NO production, sub-G1 accumulation, upregulation of LncRNA GAS5, and activation of caspase-8 and caspase-9 [131]. The methylene chloride fraction of *Vernonia leopoldii* triggered apoptosis and autophagic cell death in HepG2 and MCF-7 cells [132]. Again, both aqueous root and leaf extracts of *Jurinea dolomiaea* caused nuclear condensation and fragmentation characteristic of apoptotic cell death in Ehrlich ascites carcinoma cells [133]. Combination treatment with *Vernonia amygdalina* and cisplatin produced synergistic anticancer activity characterized by induced apoptosis in PANC-1 cells accompanied by p53 upregulation and Bcl-2 suppression [134]. *Cichorium endivia* root extract also promoted apoptosis through regulation of apoptotic genes [135], whereas nanoemulsions prepared from *Cichorium intybus* extract triggered mitochondrial membrane depolarization and ROS-mediated apoptosis in MDA-MB-231 cells [136].

Methanolic extracts of *Artemisia herba-alba* induced apoptosis in colorectal cancer cells while suppressing PI3K/Akt/mTOR signaling. Various phytochemicals such as β-eudesmol (**15**, Figure 2), ephedrine (**45**), hydroxyflavone (**46**), quinolinic acid (**47**), 4-hydroxybenzoic acid (**48**), borneol (**49**), and camphor (**50**) (Figure 3) are the major constituents of *A. herba-alba* methanolic extract [137]. *Helichrysum sanguineum* solvent extracts selectively induced apoptosis in MCF-7 cells without harming normal fibroblasts [138]. Likewise, *Artemisia judaica* extract containing benzamide (**51**), oxadiazine (**52**), benzofuran-carboxylic acid (**53**), and pyrimidine (**54**) derivatives (Figure 3) caused ROS accumulation, mitochondrial dysfunction, caspase-3 activation, and mitochondria-dependent apoptosis [139].

Ethyl acetate extracts of *Sphaeranthus amaranthoides* induced mitochondrially mediated apoptosis through disruption of the Bcl-2/Bax balance and activation of caspases-9 and -3 in AGS and HT-29 cells. Major constituents of these ethyl acetate extracts include methyl 10,13-dimethyltetradecanoate (**56**), 3,7,11,15-tetramethyl-2-hexadecen-1-ol (**57**), and 1,3,5-triazine (**58**) derivatives (Figure 3) [140]. Ethanolic extracts of *Artemisia fragrantissima* also promoted apoptosis in A549 lung cancer cells via caspase-3 upregulation and Bcl-2 downregulation [141].

Nanoparticles synthesized using *Artemisia turcomanica* extract induced apoptosis in AGS cells through upregulation of BAX, CASP3, and CASP9, with concomitant suppression of BCL2 [142]. Other similer apoptotic effects were observed for *Adenoon indicum* extracts in HT-29 colorectal cells [139] and *Tanacetum vulgare* fractions in HT-29 cells and Ehrlich tumor-bearing mice [143].

A mixture of *Elephantopus scaber* and *Phaleria macrocarpa* (Thymelaeaceae) leaf extracts enhanced apoptosis in T47D breast cancer cells by suppressing p-Nrf2, p-ERα, and PI3K/AKT/mTOR pathways. In silico studies further suggested that growth in T47D cells occurred through inhibition of ERα and the Nrf2 complex, by disrupting the crosstalk among these key signaling pathways [144]. *Taraxacum officinale* extracts increased mitochondrial permeability, cytochrome c release, ROS generation, and apoptosis in MCF-7 cells [145], while *Echinops shakrokii* extracts activated caspase-3 and promoted immune-mediated antitumor activity [146]. The aqueous extract of *Achillea biebersteinii* induced early apoptosis in ependymal tumor cells while suppressing TRPC6 and NF-κB signaling [147]. *Anacyclus pyrethrum* extract activated intrinsic apoptosis via caspase-3 and caspase-9 upregulation and Bcl-2 downregulation in A549 cells [148]. *Taraxacum officinale* also induced apoptosis in lung cancer cells through G1/S arrest [149].

The methanolic extracts from *Centaurea* species increased apoptotic indices and activated caspase-3, cleaved PARP, and p53 in HT-29 cells [150]. Both methanol and dichloromethane extracts of *Centaurea lycaonica* induced mitochondrial apoptosis through an increased Bax/Bcl-2 ratio and elevated caspase-3/-9 activity [151]. The ethanolic extract of *Ageratum fastigiatum* increased the sub-G0/G1 population and reduced migration and colony formation in MDA-MB-231 cells [152]. Subfractions from *Vernonia amygdalina* leaf extracts induced early and late apoptosis in Hs578T breast cancer cells [153]. *Artemisia absinthium* extract promoted apoptosis and necrosis in HepG-2 cells while inhibiting PI3K/Akt/mTOR signaling [154]. *Gundelia tournefortii* water extract enhanced apoptosis through BAX upregulation and HSP90 suppression in A549 lung cancer cells. The *G. tournefortii* water extract contains major bioactive compounds, namely chlorogenic acid (**44**), caffeic acid (**59**), epicatechin (**60**), and apigenin (**61**) [155]. The essential oil (EO) of *Conyza bonariensis* containing cis-lachnophyllum ester (**22**, Figure 2) as the bioactive compound induced ROS-mediated apoptosis in SK-MEL-28 melanoma cells and modulated ERK1/2, JNK, p38 MAPK, NF-κB, and PKB/AKT signaling [80,156]. Finally, flower EO of *Acmella paniculata* containing δ-amorphene (**62**), limonene (**63**), spathulenol (**64**), E-nerolidol (**65**), and β-bisabolene(**66**) (Figure 3) exhibited strong apoptosis-inducing activity in SCC9 oral cancer cells, with compound **64** showing strong interaction with p53 protein in molecular docking analyses [157].

#### 5.4.2. Cell Cycle Arrest Induced by Asteraceae Plant Extracts

Many plant extracts exerted anticancer effects through interference with cell cycle progression at different checkpoints. *Vernonia leopoldii* extracts induced G2/M arrest in HepG2 and MCF-7 cells [132]. Combined treatment of *Vernonia amygdalina* with cisplatin caused G1 arrest in PANC-1 cells together with cyclin D1 suppression [134]. *Cichorium endivia* root extract induced S-phase arrest [135], while *Cichorium intybus* nanoemulsion caused G2/M arrest in breast cancer cells [136]. *Artemisia herba-alba* extract downregulated CDK1 and cyclin B1, resulting in G2/M arrest [137]. *Helichrysum sanguineum* extracts also caused cell-cycle arrest in MCF-7 cells [138]. *Sphaeranthus amaranthoides* extract induced G0/G1 arrest through regulation of p21, p27, cyclin D1, and CDK4 [140].

*Artemisia fragrantissima* caused G2/M arrest in A549 cells [141], while *Artemisia turcomanica*-derived nanoparticles induced sub-G1 arrest [142]. *Adenoon indicum* extract promoted S-phase accumulation and partial G2/M arrest in HT-29 cells [158]. The *Tanacetum vulgare* fraction caused genotoxic stress-associated cell-cycle arrest [143]. Extract combinations of Elephantopus scaber and Phaleria macrocarpa also induced cell-cycle arrest through simultaneous inhibition of PI3K/AKT/mTOR, ERα, and Nrf2 signaling [144].

*Taraxacum officinale* induced G1 arrest in MCF-7 cells [145] and G1/S arrest in A549 cells [146]. *Achillea biebersteinii* extract promoted G0/G1 arrest in ependymal tumor cells [147], whereas *Anacyclus pyrethrum* caused G1-phase arrest in A549 cells [148]. Subfractions of *Vernonia amygdalina* inhibited G2/M progression in Hs578T cells [153], and *Artemisia absinthium* caused accumulation in sub-G1 and G1 phases with reduction of S and G2/M populations [154].

#### 5.4.3. ROS Generation and Oxidative Stress Induced by Asteraceae Plant Extracts

Reactive oxygen species (ROS)-mediated cytotoxicity represented another major anticancer mechanism. Nanoemulsions of *Cichorium intybus* increased ROS accumulation and mitochondrial depolarization in MDA-MB-231 cells [136]. *Artemisia judaica* induced ROS accumulation associated with mitochondrial dysfunction and apoptosis [139]. *Taraxacum officinale* extract elevated intracellular ROS levels comparable to doxorubicin treatment [145].

The EO of *C. bonariensis* significantly increased ROS production in melanoma cells, and antioxidant pretreatment reduced cytotoxicity, confirming ROS-dependent activity [156]. In addition, *C. bonariensis* EO modulated oxidative stress-related enzymes in zebrafish embryos [156]. ROS-associated apoptosis was also implicated in the activity of *Achillea multifida* [130] and *Artemisia herba-alba* [137].

#### 5.4.4. PI3K/Akt/mTOR Pathway Inhibition Induced by Asteraceae Plant Extracts

Suppression of the PI3K/Akt/mTOR signaling pathway emerged as a common target associated with inhibition of proliferation and survival. Combined treatment with *Vernonia amygdalina* and cisplatin suppressed PI3K/mTOR signaling in pancreatic cancer cells [134]. *A. herba-alba* inhibited the PI3K/Akt/mTOR pathway in colorectal cancer cells [137]. The extract mixture of *Elephantopus scaber* and *Phaleria macrocarpa* simultaneously inhibited PI3K/AKT/mTOR signaling together with ERα and Nrf2 pathways [144].

Similarly, *Artemisia absinthium* downregulated PI3K/Akt/mTOR signaling at both gene and protein levels in HepG-2 cells [154]. *Conyza bonariensis* EO also modulated PKB/AKT signaling [80].

#### 5.4.5. Mitochondrial Dysfunction and Intrinsic Apoptotic Signaling Induced by Asteraceae Plant Extracts

Several studies specifically demonstrated involvement of mitochondrially mediated apoptosis. *Cichorium intybus* nanoemulsion induced mitochondrial membrane depolarization [136]. *A. judaica* caused mitochondrial dysfunction and caspase-3 activation [139]. *S. amaranthoides* disrupted mitochondrial apoptotic balance via Bcl-2/Bax modulation and caspase activation [140]. *T. officinale* enhanced mitochondrial membrane permeability and cytochrome c release [145]. *C. lycaonica* extracts activated the mitochondrial apoptotic pathway through an increased Bax/Bcl-2 ratio and caspase activation [151].

#### 5.4.6. Caspase Activation Induced by Asteraceae Plant Extracts

Activation of executioner and initiator caspases was widely associated with apoptosis induction. *A. sieberi* nanoparticles increased caspase-8 and caspase-9 activity [131]. *Artemisia judaica* activated caspase-3 [139], while *S. amaranthoides* stimulated caspases-9 and -3 [140]. *Artemisia fragrantissima* upregulated caspase-3 [141]. *Artemisia turcomanica* nanoparticles increased CASP3 and CASP9 expression [142]. *Echinops shakrokii* induced apoptosis via caspase-3 activation [146]. *Anacyclus pyrethrum* enhanced caspase-3 and caspase-9 levels [148]. *Centaurea* species also activated caspase-3 and cleaved PARP [150], while *Centaurea lycaonica* elevated caspase-3/-9 activity [151].

#### 5.4.7. Modulation of Bcl-2 Family Proteins by Asteraceae Plant Extracts

Regulation of pro- and anti-apoptotic Bcl-2 family proteins was commonly observed. *Vernonia amygdalina* combined with cisplatin downregulated Bcl-2 [134]. *S. amaranthoides* altered the Bcl-2/Bax balance [140]. *Artemisia fragrantissima* decreased Bcl-2 expression [141]. *Artemisia turcomanica* nanoparticles suppressed BCL2 while increasing BAX [142]. *Anacyclus pyrethrum* downregulated Bcl-2 [148], *Centaurea lycaonica* increased the Bax/Bcl-2 ratio [151], and *Gundelia tournefortii* enhanced BAX expression while suppressing HSP90 [155].

#### 5.4.8. Modulaton of NF-κB, MAPK, and Other Signaling Targets by Asteraceae Plant Extracts

Several extracts targeted additional oncogenic signaling pathways. *Achillea biebersteinii* suppressed TRPC6 and NF-κB expression [147]. *Conyza bonariensis* EO modulated ERK1/2, JNK, p38 MAPK, NF-κB, and PKB/AKT pathways [80]. Molecular docking studies further supported interactions of compound **22** with ERK1, JNK1, p38 MAPK, NF-κB, and AKT proteins [80]. The extract combination of *Elephantopus scaber* and *Phaleria macrocarpa* inhibited p-Nrf2 and p-ERα signaling [144], while *Acmella paniculata* constituents showed strong interaction with p53 [157].

#### 5.4.9. Anti-Migration, Anti-Colony Formation, and Anti-Metastatic Effects of Asteraceae Plant Extracts

Some studies highlighted inhibition of cancer-cell migration and colony formation. *Vernonia leopoldii* inhibited migration of hepatocellular carcinoma cells [132]. *Centaurea* species suppressed migration and colony formation in HT-29 cells [150]. *Ageratum fastigiatum* inhibited migration and colony formation in both 2D and 3D models [152]. EO from *Acmella paniculata* also reduced orosphere formation in oral cancer cells [157].

#### 5.4.10. Immunomodulatory and Anti-Inflammatory Effects of Asteraceae Plant Extracts

Additional anticancer mechanisms included immunomodulation and anti-inflammatory activity. *Cichorium endivia* extract showed anti-inflammatory activity through cytokine suppression and selective COX-2 inhibition [135]. *Adenoon indicum* demonstrated anti-inflammatory effects alongside apoptosis induction [158]. *Echinops shakrokii* enhanced lymphocyte proliferation and macrophage activity, contributing to tumor regression in vivo [146].

Overall, the studies reviewed demonstrate that Asteraceae-derived plant extracts exert potent anticancer effects through multiple interconnected mechanisms targeting tumor cell survival, proliferation, oxidative balance, and metastatic behavior (as summarized in Table 4). Apoptosis induction emerged as the predominant mechanism, particularly through activation of the intrinsic mitochondrial pathway involving modulation of Bax/Bcl-2 balance, cytochrome c release, caspase activation, and mitochondrial membrane depolarization. Many extracts also simultaneously induced cell-cycle arrest at different checkpoints, indicating coordinated suppression of proliferation and promotion of apoptotic death. ROS-mediated oxidative stress was another recurring mechanism closely linked to mitochondrial dysfunction and apoptosis, while inhibition of major oncogenic pathways such as PI3K/Akt/mTOR, NF-κB, MAPK, ERα, and Nrf2 further highlighted the multitargeted nature of these phytochemicals. In addition to direct cytotoxicity, several extracts demonstrated anti-metastatic, anti-inflammatory, and immunomodulatory activities, suggesting broader therapeutic potential against cancer progression and tumor-associated inflammation. The incorporation of nanoformulations and biosynthesized nanoparticles further enhanced anticancer efficacy in some studies by improving cellular uptake and apoptotic signaling. Despite these promising findings, most studies remain limited to in vitro models, and further investigations involving standardized extracts, isolation of active compounds, pharmacokinetic profiling, toxicity evaluation, and well-designed in vivo and clinical studies are necessary to validate the therapeutic potential of Asteraceae plants as sources of novel anticancer agents and adjuvant therapies.

### 5.5. Anticancer Mechanisms/Targets of Bioactive Phytochemicals from Asteraceae Species

The studies mentioned above examined plant extracts from various Asteraceae species and identified their components using different techniques, but did not assess them individually. In contrast, the studies below isolated bioactive compounds from Asteraceae species and evaluated their therapeutic properties separately.

#### 5.5.1. Apoptosis Induction by Asteraceae-Derived Bioactive Phytochemicals 

Induction of apoptosis was the predominant anticancer mechanism reported for the isolated phytochemicals. Dehydrozaluzanin C (**67**, Figure 3) isolated from *Ainsliaea macrocephala* induced apoptosis in HT-29 and HCT-116 colon cancer cells through downregulation of Bcl-2 and upregulation of Bax and cleaved caspase-3 [159]. Among the bioactive constituents identified from *Gerbera piloselloides*, cyclobrachycoumarin (**68**, Figure 3) also triggered dose-dependent apoptosis in colorectal cancer cells, accompanied by increased cleaved PARP expression [160]. Herniarin (**69**, Figure 3) from *Artemisia dracunculus* activated caspase-mediated apoptosis in bladder cancer cell lines and modulated pro- and anti-apoptotic proteins [161]. Centrapalus coumarin F (**70**, Figure 3) isolated from *Centrapalus pauciflorus* induced apoptosis in HPV-18-positive HeLa cervical cancer cells while suppressing migration and invasion [162]. Chlorojanerin (**71**, Figure 3) from *Centaurothamnus maximus* triggered apoptosis in A549 lung cancer cells, supported by modulation of apoptosis-related genes and effective binding to Bcl-2 [163]. Eupalan A (**72**, Figure 3) isolated from *Eupatorium chinense* promoted apoptosis through increased Bax and decreased Bcl-2 expression [164].

Alantolactone (**73**, Figure 3) from *Inula helenium* induced apoptosis and pyroptosis in anaplastic thyroid cancer cells, evidenced by PARP cleavage [165]. Cyna ropicrin-3-O-4-hydroxymethacrylate (**74**, Figure 3) isolated from *Himalaiella heteromalla*, promoted apoptosis, disrupted mitochondrial function, and downregulated Bcl-2 in pancreatic cancer cells [166]. Gaillardin (**75**, Figure 3) from *Inula oculus-christi* induced apoptosis in HL-60 leukemia cells through ROS generation [167]. Apigetrin (**76**, Figure 3) isolated from *Matricaria chamomilla* triggered apoptosis in HepG2 liver cancer cells primarily via the extrinsic apoptotic pathway, characterized by increased FasL, cleaved caspase-8, caspase-3, and PARP [168]. Taraxasterol (**77**, Figure 3) from *Taraxacum officinale* promoted apoptosis in lung cancer cells through increased Bax, caspase-9, and PARP1 expression, reduced Bcl-2 levels, and mitochondrial membrane depolarization [169]. Echinocystic acid (**78**, Figure 3) from *Eclipta prostrata* also promoted apoptosis in A549 xenograft models [170].

#### 5.5.2. Cell Cycle Arrest Induced by Asteraceae-Derived Bioactive Phytochemicals

Several phytochemicals exerted antiproliferative effects through arrest of the cell cycle at distinct phases. Dehydrozaluzanin C (**67**) induced S-phase arrest in colon cancer cells through downregulation of CDK2 and cyclin A2 [159]. Cyclobrachycoumarin (**68**) caused cell cycle arrest associated with suppression of cyclin D1 and CDK1 [160].

Herniarin (**69**) induced phase-specific arrest depending on the bladder cancer cell line: G1/S arrest in RT-112 cells, G2/M arrest in HTB9 cells, and S-phase arrest in HT1376 cells [161]. Chlorojanerin (**71**) triggered G2/M arrest in A549 cells [163], while rupicolin A (**79**, Figure 3) and rupicolin B (**80**, Figure 3) isolated from *Achillea grandifolia* also induced G2/M arrest in glioblastoma cells [171]. Eupalan A (**72**) promoted G0/G1 arrest via upregulation of p21 and downregulation of CDK4 and cyclin D [164]. Lappaol F (**81**, Figure 3) from Arctium lappa induced S-phase arrest in colon cancer cells through upregulation of CDKN1C/p57 [172].

Alantolactone (**73**) caused G2/M arrest in anaplastic thyroid cancer cells through suppression of cyclin B1 and CDC2 [165]. Compound **74** induced G2/M arrest in pancreatic cancer cells [166]. Gaillardin (**75**) caused G1 arrest in HL-60 leukemia cells [167]. Apigetrin (**76**) induced G2/M arrest through modulation of cyclin B1 and CDK1 [168]. Taraxasterol (**77**) promoted S-phase arrest in lung cancer cells [169]. Compound **82**, shown in Figure 3 and isolated from *Centaurea bruguieriana*, induced G0/G1 arrest in glioblastoma cells [173], whereas isocnicin (**83**, Figure 3) caused S and G2/M arrest and enhanced G2/M accumulation when combined with radiotherapy [174]. Echinocystic acid (**78**) induced G1-phase arrest in A549 cells [170].

#### 5.5.3. Modulation of Bcl-2 Family Proteins by Asteraceae-Derived Bioactive Phytochemicals

Regulation of pro- and anti-apoptotic Bcl-2 family proteins was a recurring mechanism. Dehydrozaluzanin C (**67**) downregulated Bcl-2 and increased Bax expression [159]. Herniarin (**69**) altered pro- and anti-apoptotic protein expression [161]. Chlorojanerin (**71**) showed strong binding affinity toward Bcl-2, supporting apoptosis induction [163].

Eupalan A (**72**) increased Bax while decreasing Bcl-2 expression [164]. Compound **74** downregulated Bcl-2, as supported by molecular docking and Western blot analyses [166]. Taraxasterol (**77**) increased Bax and reduced Bcl-2 expression in lung cancer cells [169].

#### 5.5.4. Caspase Activation and PARP Cleavage by Asteraceae-Derived Bioactive Phytochemicals

Activation of caspases and PARP cleavage contributed significantly to phytochemical-induced apoptosis. Dehydrozaluzanin C (**67**) increased cleaved caspase-3 expression [154]. Cyclobrachycoumarin (**68**) enhanced cleaved PARP levels [160]. Herniarin (**69**) activated caspase-mediated apoptosis [161].

Alantolactone (**73**) induced PARP cleavage together with apoptosis and pyroptosis [165]. Apigetrin (**76**) activated the extrinsic apoptotic pathway through increased cleaved caspase-8, caspase-3, and PARP [168]. Taraxasterol (**77**) elevated caspase-9 and PARP1 expression [169].

#### 5.5.5. ROS Generation and Oxidative Stress Induced by Asteraceae-Derived Bioactive Phytochemicals

Reactive oxygen species (ROS)-mediated cytotoxicity was reported for several compounds. Cyclobrachycoumarin (**68**) increased intracellular ROS levels in colorectal cancer cells [160]. Gaillardin (**75**) promoted ROS generation in HL-60 leukemia cells, contributing to apoptosis induction [167].

#### 5.5.6. PI3K/Akt/mTOR Pathway Inhibition by Asteraceae-Derived Bioactive Phytochemicals

Suppression of the PI3K/Akt/mTOR pathway was identified as an important anticancer target. Echinocystic acid (**78**) inhibited PI3K/Akt/mTOR signaling in A549 lung cancer cells while upregulating Par3 expression [170]. Eupalan A (**72**) inhibited the DNA-PK/AKT/p53 signaling axis, resulting in p21 activation and cell cycle arrest [164].

#### 5.5.7. PPARγ Activation by Asteraceae-Derived Bioactive Phytochemicals

Activation of PPARγ was specifically identified as the primary target of dehydrozaluzanin C (**67**). Compound **67** enhanced PPARγ transcriptional activity, and inhibition or silencing of PPARγ reversed its antiproliferative and proapoptotic effects, demonstrating that PPARγ activation is central to its anticancer mechanism [159].

#### 5.5.8. Regulation of Cell Cycle Proteins by Asteraceae-Derived Bioactive Phytochemicals

Several phytochemicals directly modulated cyclins, cyclin-dependent kinases (CDKs), and related regulatory proteins. Dehydrozaluzanin C (**67**) suppressed CDK2 and cyclin A2 [159]. Cyclobrachycoumarin (**68**) decreased cyclin D1 and CDK1 expression [160]. Eupalan A (**72**) increased p21 while reducing CDK4 and cyclin D levels [164].

Lappaol F (**81**) upregulated CDKN1C/p57, which mediated its S-phase arrest effects [172]. Alantolactone (**73**) reduced cyclin B1 and CDC2 expression [165]. Apigetrin (**76**) regulated cyclin B1 and CDK1 [168].

#### 5.5.9. Inhibition of Migration, Invasion, EMT, and Metastatic Potential by Asteraceae-Derived Bioactive Phytochemicals

Several compounds exhibited antimetastatic effects by suppressing migration, invasion, and EMT. Herniarin (**69**) inhibited migration of bladder cancer cells [168]. Centrapalus coumarin F (**70**) suppressed migration and invasion in cervical cancer cells and also demonstrated antiestrogenic activity [162].

Apigetrin (**76**) reduced colony formation and migration in HepG2 cells [168]. Taraxasterol (**77**) inhibited migration by suppressing epithelial–mesenchymal transition (EMT) [169]. Echinocystic acid (**78**) reduced migration and invasion in lung cancer cells [166]. Compound **82** also reduced glioblastoma cell migration [173].

#### 5.5.10. Signaling Pathway Modulation by Asteraceae-Derived Bioactive Phytochemicals

Several phytochemicals targeted additional signaling pathways involved in cancer progression. Herniarin (**69**) modulated the Erk signaling pathway [161]. Eupalan A (**72**) inhibited DNA-PK/AKT/p53 signaling [164]. Echinocystic acid (**78**) suppressed PI3K/Akt/mTOR signaling and enhanced Par3 expression [170].

#### 5.5.11. Mitochondrial Dysfunction and Intrinsic Apoptosis Induced by Bioactive Phytochemicals

Mitochondrial dysfunction was involved in the anticancer activity of some phytochemicals. Compound **74** disrupted mitochondrial function in pancreatic cancer cells [166]. Taraxasterol (**77**) reduced mitochondrial membrane potential and activated intrinsic apoptotic signaling [169].

#### 5.5.12. Modulation of the Immune Response and Tumor Microenvironment by Asteraceae-Derived Bioactive Phytochemicals

Taraxasterol (**77**) exhibited immunomodulatory activity by reducing regulatory T (Treg) cells and increasing CD107a^+^ NK-cell activity, thereby modulating the tumor microenvironment and enhancing antitumor immunity [169].

#### 5.5.13. Radiosensitization and Combination Therapy by Asteraceae-Derived Bioactive Phytochemicals

Some phytochemicals enhanced the effects of conventional therapies. Isocnicin (**83**) synergistically enhanced the antiproliferative effects of radiotherapy in glioblastoma cells, significantly increasing G2/M accumulation after irradiation [174]. In contrast, compound **82** exhibited antagonistic effects when combined with radiotherapy, suggesting that it may be more beneficial after radiation treatment rather than concurrently [173].

Gaillardin (**75**) enhanced the sensitivity of HL-60 leukemia cells to arsenic trioxide treatment, indicating its potential utility in combination therapy for acute promyelocytic leukemia [167].

The findings summarized in this section demonstrate that isolated phytochemicals from Asteraceae species possess diverse and potent anticancer activities mediated through multiple molecular targets and signaling pathways (summarized in Table 5). Similar to the crude extracts discussed previously, apoptosis induction emerged as the principal mechanism of action, predominantly involving mitochondrial dysfunction, modulation of Bax/Bcl-2 balance, activation of caspases, and PARP cleavage. However, the use of purified compounds provided clearer mechanistic insights into specific molecular interactions, such as the activation of PPARγ by dehydrozaluzanin C, inhibition of DNA-PK/AKT/p53 signaling by Eupalan A, and suppression of PI3K/Akt/mTOR signaling by echinocystic acid. These findings highlight the advantage of studying isolated compounds in identifying precise intracellular targets and pathways responsible for anticancer effects. In addition to apoptosis, many phytochemicals induced cell-cycle arrest at G0/G1, S, or G2/M phases through regulation of cyclins, CDKs, p21, p57, and related checkpoint proteins, thereby inhibiting cancer-cell proliferation. ROS-mediated oxidative stress also contributed significantly to cytotoxicity in several compounds, suggesting that oxidative imbalance serves as an important upstream trigger of apoptosis and mitochondrial collapse. Notably, several phytochemicals demonstrated anti-metastatic activities by suppressing migration, invasion, epithelial–mesenchymal transition, and colony formation, indicating their potential utility in preventing tumor progression and dissemination. Some compounds further exhibited immunomodulatory effects and enhanced sensitivity to radiotherapy or chemotherapeutic agents, emphasizing their potential role as adjuvant anticancer agents in combination therapy. Compared with crude extracts, isolated phytochemicals provide better opportunities for mechanistic characterization, structure–activity relationship analysis, dose standardization, and drug development. Nevertheless, most studies remain confined to in vitro investigations, with limited in vivo validation and scarce clinical evidence. Additional studies focusing on pharmacokinetics, toxicity, bioavailability, molecular docking validation, and translational models are therefore necessary to fully establish the therapeutic potential of Asteraceae-derived phytochemicals as candidates for novel anticancer drugs.

### 5.6. Structure–Activity Relationships (SAR) of Major Asteraceae-Derived Phytochemicals

Despite the structural diversity of Asteraceae-derived phytochemicals, several recurring SAR patterns emerge across the major chemical classes, particularly sesquiterpene lactones, flavonoids, phenolic acids, terpenoids, coumarins, and lignans. These structural features strongly influence anticancer, anti-inflammatory, and antioxidant activities through modulation of signaling pathways, apoptosis regulators, oxidative stress, and kinase-mediated processes.

#### 5.6.1. Sesquiterpene Lactones

Sesquiterpene lactones represent one of the most pharmacologically active classes in Asteraceae species. Their biological activity is largely attributed to the presence of the α-methylene-γ-lactone moiety, a highly electrophilic structural feature capable of forming covalent bonds with nucleophilic thiol groups in proteins, particularly cysteine residues in transcription factors and signaling enzymes. This interaction contributes to inhibition of NF-κB, PI3K/Akt, MAPK, and other cancer-related signaling pathways.

Compounds such as dehydrozaluzanin C (**67**) [159], chlorojanerin (**71**) [163], alantolactone (**73**) [165], gaillardin (**75**) [167], and the guaianolides isolated from *Himalaiella heteromalla* [166] demonstrated potent antiproliferative and pro-apoptotic effects associated with apoptosis induction, ROS generation, and cell cycle arrest. Increased lipophilicity and the presence of epoxide or hydroxyl substituents appear to enhance membrane permeability and target interactions, thereby improving cytotoxic potency. Moreover, sesquiterpene lactones with conjugated carbonyl systems generally exhibit stronger activity due to enhanced electrophilic reactivity toward intracellular targets.

#### 5.6.2. Flavonoids and Flavonoid Glycosides

Flavonoids exhibit broad anticancer and antioxidant activities through modulation of NF-κB, MAPK, PI3K/Akt/mTOR, and Nrf2 signaling pathways. Their activity is strongly influenced by hydroxylation patterns, methoxylation, glycosylation, and conjugation within the flavonoid backbone.

Hydroxyl groups, particularly in the B-ring, enhance radical scavenging ability and contribute to antioxidant activity through stabilization of reactive oxygen species [175,176,177,178]. Compounds such as apigetrin (**76**) [168], luteolin derivatives, and methoxyflavones isolated from *Centaurea bruguieriana* [173] demonstrated apoptosis induction and cell cycle arrest. In general, aglycone flavonoids exhibit stronger cytotoxic and anti-inflammatory activity than glycosylated forms because glycosylation may reduce lipophilicity and cellular uptake [179,180]. Conversely, methoxylation tends to improve membrane permeability and metabolic stability, potentially enhancing bioavailability and anticancer activity [181].

Structure-dependent modulation of signaling proteins also appears important. For example, methoxylated flavonoids demonstrated stronger effects on glioblastoma cell cycle regulation [173], whereas hydroxyl-rich flavonoids were more closely associated with antioxidant and ROS-modulating activities.

#### 5.6.3. Phenolic Acids

Phenolic acids, including chlorogenic acid (**44**) and caffeic acid (**59**), primarily contribute antioxidant and cytoprotective effects through ROS scavenging and activation of Nrf2-mediated antioxidant responses [155]. Their activity is largely determined by the number and position of hydroxyl groups attached to the aromatic ring.

Compounds containing catechol structures generally exhibit stronger antioxidant activity because adjacent hydroxyl groups enhance electron donation and free radical stabilization [180,182]. However, compared with sesquiterpene lactones and terpenoids, phenolic acids often demonstrate lower direct cytotoxicity against cancer cells. Instead, they may contribute synergistically within crude extracts by modulating oxidative stress, inflammation, and mitochondrial dysfunction.

#### 5.6.4. Triterpenoids

Terpenoids and triterpenoids exhibit diverse anticancer mechanisms depending on their structural complexity and degree of functionalization. Compounds such as taraxasterol (**77**) [169] and echinocystic acid (**78**) [170] demonstrated apoptosis induction, EMT suppression, and inhibition of PI3K/Akt/mTOR signaling.

Hydrophobic pentacyclic structures enhance membrane interactions and intracellular accumulation, while hydroxyl and carboxyl functional groups contribute to modulation of signaling proteins and mitochondrial pathways. Triterpenoids possessing planar hydrophobic skeletons generally exhibit stronger anti-metastatic and anti-inflammatory activities due to improved interactions with membrane-associated targets and kinase domains. Structural modifications that increase polarity may improve solubility but sometimes reduce membrane permeability and intracellular potency.

#### 5.6.5. Coumarins and Related Aromatic Compounds

Coumarin derivatives isolated from *Gerbera piloselloides* [160] and herniarin (**69**) from *Artemisia dracunculus* [161] demonstrated apoptosis induction, ROS generation, and cell cycle arrest. SAR trends suggest that methoxy and hydroxyl substitutions on the coumarin scaffold significantly influence cytotoxic activity and kinase interactions. Increased aromatic conjugation may also enhance DNA intercalation and oxidative stress induction.

Similarly, meroterpenoids such as centrapalus coumarin F (**70**) [162] demonstrated potent anticancer and anti-migratory effects, likely due to the combined pharmacophoric features of both terpenoid and coumarin moieties, which may facilitate simultaneous modulation of multiple molecular targets.

#### 5.6.6. Lignans and Other Polyphenolic Compounds

Lignans such as lappaol F (**81**) [172] exhibited significant antiproliferative activity through modulation of cell cycle proteins, particularly CDKN1C/p57. Their polyphenolic structures enable interactions with signaling proteins involved in proliferation and apoptosis. Increased hydroxylation and stereochemical orientation may influence binding affinity toward regulatory enzymes and transcription factors [183,184].

Overall, polyphenolic compounds tend to exhibit multitarget activities through combined antioxidant, pro-apoptotic, and signaling-modulatory effects, although their potency is often lower than highly electrophilic sesquiterpene lactones.

These observations indicate that the pharmacological activities of Asteraceae-derived compounds are strongly dependent on specific structural features, highlighting the importance of rational structural optimization for future drug development and translational applications.

## 6. Conclusions and Future Directions

Plant species from the Asteraceae family represent a rich source of therapeutic agents with significant pharmacological activities across inflammation, cancer, and oxidative stress. Evidence from in vitro, in vivo, and in silico research reveals that these plants produce multi-target effects through modulation of important biochemical pathways, including NF-κB, MAPK, PI3K/Akt, and Nrf2 signaling. Their ability to simultaneously decrease pro-inflammatory mediators, increase antioxidant defense, and modulate cancer-related processes such as apoptosis and cell proliferation underscores their usefulness as integrative therapeutic agents. Importantly, the overlap between antioxidant, anti-inflammatory, and anticancer mechanisms underscores a systems-level mode of action, which may offer advantages over single-target drugs. Overall, Asteraceae species provide a strong pharmacological foundation for the development of novel, plant-based therapeutics.

Future research should prioritize the systematic identification and prioritization of lead compounds with favorable efficacy, safety, and pharmacokinetic properties for further preclinical and clinical development. Detailed phytochemical characterization and standardization of extracts are essential to ensure reproducibility, facilitate cross-study comparisons, and support the establishment of reporting standards for mechanistic botanical research. More comprehensive research on molecular mechanisms and therapeutic targets is needed to clarify structure–activity relationships and identify the most potent bioactive constituents. Advances in drug delivery systems, such as nanoparticles and nanoemulsions, should be further explored to enhance bioavailability and target specificity. Investigating synergistic interactions between Asteraceae compounds and conventional drugs may also improve therapeutic outcomes, particularly in cancer treatment. Lastly, greater emphasis should be placed on well-designed in vivo studies and clinical trials to validate efficacy, establish safety profiles, and accelerate the translation of promising Asteraceae-derived compounds into evidence-based therapeutic applications.

## Figures and Tables

**Table 1:** Medicinal plant species from the Asteraceae family traditionally used to manage infections, inflammation, and oxidative stress related-conditions.

Plant Species	Part(s) Used	Traditional Medicinal Uses	Reference
*Acilepis saligna*	Leaves, bark, roots	Treatment of fever, rheumatism, and skin disorders	[13]
*Achillea millefolium*	Leaves	Gastrointestinal disorders, respiratory infections, wound healing, and skin inflammation	[27]
*Achillea tenuifolia*	Fruits, leaves	Treatment of hemorrhage, pneumonia, rheumatic pain, and wounds	[28]
*Achillea arabica*	Whole plant	Rheumatic pain, fever, respiratory infections, gastrointestinal disorders, and wound healing	[29,30]
*Ageratum conyzoides*	Leaves, twigs	Treatment of infections and wounds	[31]
*Artemisia abyssinica*	Leaves	Management of fever and respiratory infections	[32]
*Anthemis odontostephana*	Leaves	Eye pain, burns, colds, malaria, headache, and liver cleansing	[33]
*Artemisia absinthium*	Aerial parts	Indigestion, abdominal pain, laxative use, and intestinal parasites	[34,35]
*Bidens pilosa*	Leaves	Treatment of poisoning, wounds, and infections	[36]
*Baccharis anthelmintica*	Seeds, leaves	Management of diabetes, asthma, paralysis, and phlegmatic conditions	[37]
*Carthamus tinctorius*	Leaves, roots	Treatment of rheumatism and osteoporosis	[38]
*Chromolaena odorata*	Leaves	Wounds, burns, skin infections, malaria, fever, and fungal infections	[39]
*Chiliadenus glutinosus*	Leaves, roots	Gastrointestinal disorders, respiratory infections, hypertension, and rheumatic pain	[40,41]
*Chrysophthalmum montanum*	Aerial parts	Wound healing and injury management	[42]
*Eclipta alba*	Leaves, shoots	Diarrhea, ulcers, hemorrhage, liver and spleen disorders	[43]
*Eclipta prostrata*	Leaves, stems, whole plant	Wounds, burns, ulcers, dermatitis, and snakebites	[44]
*Emilia sonchifolia*	Leaves	Treatment of diarrhea	[45]
*Emilia praetermissa*	Leaves	Fever, sore throat, malaria, asthma, and epilepsy	[46]
*Echinops ritrodes*	Whole plant, fruits	Respiratory and digestive disorders	[47,48]
*Echinops kebericho*	Roots	Headache, toothache, and vomiting	[49]
*Eremanthus erythropappus*	Leaves	Pain relief, infections, ulcers, malaria, and jaundice	[50]
*Guizotia abyssinica*	Seeds, roots	Cough and stomachache	[51]
*Gundelia tournefortii*	Leaves	Digestive aid, antiparasitic, liver tonic, and antibiotic use	[52,53]
*Helichrysum leucocephalum*	Whole plant	Colds, liver disorders, and skin infections	[54,55]
*Launaea intybacea*	Leaves	Edible plant used for pain and fever relief	[56]
*Matricaria pubescens*	Leaves, flowers, roots	Cough, kidney disorders, rheumatism, and analgesic use	[57]
*Matricaria aurea*	Leaves	Bronchitis, sore throat, tonsillitis	[58]
*Onopordum tauricum*	Leaves	Liver disorders	[42]
*Onopordum acanthium*	Leaves	Fever and kidney disorders	[59]
*Platycarpha glomerata*	Leaves, roots	Wounds, burns, joint disorders, eye problems, and cancer	[60]
*Silybum marianum*	Whole plant	Hepatoprotective, galactogenic, digestive, and antidiabetic uses	[61]
*Tanacetum parthenium*	Leaves, roots	Headache, migraine, inflammation, and joint pain	[62]
*Xanthium strumarium*	Leaves	Skin and fungal infections	[63]
*Vernonia amygdalina*	Leaves, roots	Gastrointestinal disorders, malaria, inflammation, and infections	[64]

**Table 2:** Anti-inflammatory effect of plant extracts from Asteraceae species, highlighting their experimental models, molecular targets, and reported biological effects.

Plant Species	Extract(s) (Parts Used)	Model/System	Main Mechanisms/Targets	Key Findings	Ref.
*Kalimeris incisa* (Fisch.) DC. (Asteraceae)	MeOH (Aerial)	LPS-stimulated RAW 264.7 cells	NF-κB, MAPK, PI3K/Akt	↓ NO, iNOS, COX-2, TNF-α, IL-1β, IL-6; ↓ phosphorylation	[84]
*Montanoa grandiflora* (Hemsl) DC. (Asteraceae)	MeOH (Leaves)	In vitro & in vivo (TPA, LPS)	Cytokine modulation	↑ IL-10; ↓ TNF-α, IL-1β, IL-6, NO; ↓ edema	[97]
*Artemisa alba Turra* (Asteraceae)	EtOH (Aerial)	In vivo (rat model)	NF-κB, NLRP3	↓ IL-1β, IL-18, inflammasome markers	[90]
*Tagetes erecta* L. (Asteraceae)	EtOH (Flower)	In silico	TNF-α, IL-6, and iNOS	NR	[96]
*Scorzonera ketzkhowelii* (Sosn.) ex Grossh. (Asteraceae)	EtAc (Aerial)	Enzyme assays	COX inhibition	Strong COX-I/II inhibition (like indomethacin)	[92]
*Cynara cardunculus* L.	Aqueous and DCM (Leaves)	Enzyme assays	COX-2, 5-LOX	Strong enzyme inhibition	[95]
*Ixeridium dentatum* (Thunb.) Tzvelev (Asteraceae)	Aqueous (Aerial)	RAW264.7 cells	NF-κB	↓ COX-2, TNF-α, IL-6, IL-1β	[85]
*Tagetes parryi* (Gray) A. (Asteraceae)	EO (Aerial)	In vitro & in vivo (TPA, LPS)	No pathway	↓ edema, NO, TNF-α, IL-6; antinociceptive effects	[87]
*Vernonanthura tweediana* (Baker) H. Rob. (Asteraceae)	EO (Leaves)	In vivo	Cytokine regulation	↓ leukocyte migration; ↓ IL-1β, IL-6, TNF	[98]
*Atractylodes lancea* (Thunb.) DC. (Asteraceae)	Aqueous (Rhizome)	Microglial cells	Gene regulation	↓ NO, cytokines; neuroinflammatory protection	[91]
*Erigeron sublyratus* (Roxb. Ex) DC. (Asteraceae)	EO (Aerial)	LPS-stimulated RAW 264.7 cells	VEGFR-2, COX-2	↓ IL-1β, IL-18, inflammasome markers	[100]
*Artemisia scoparia* (Waldst. & Kit.) (Asteraceae)	EO (Aerial)	RAW264.7 + in silico	PPAR, NF-κB	↓ NO, TNF-α; multi-pathway targeting	[101]
*Achillea fragrantissima* (Forssk.) Sch.Bip. (Asteraceae)	EO (Aerial)	RAW264.7 cells	Cytokine suppression	↓ TNF-α, IL-2, IL-6	[99]
*Blumea lanceolaria* (Roxb.) (Asteraceae	EO (Leaves, Stems, and Roots)	In vitro & in vivo	NF-κB, MAPK	↓ NO, TNF-α, IL-6; ↓ edema	[88]
*Crossostephium chinense* (Makino) L. (Asteraceae)	EO (Leaves)	RAW264.7 cells	NF-κB, MAPK, Nrf2	↓ NO, ROS, cytokines	[89]
*Artemisia abrotanum* L. (Asteraceae)	EO (Aerial)	RAW264.7 cells	iNOS inhibition	↓ NO, iNOS expression	[102]

Note: NR = not reported. “↑” indicates upregulation or increase; “↓” indicates downregulation or decrease.

**Table 3:** Summary of anti-inflammatory activities of evaluated Asteraceae phytochemicals, highlighting their source, plant extract, experimental models, molecular mechanisms/targets and reported biological outcomes.

Evaluated Phytochemicals	Plant Species	Extracts (Parts Used)	Model/System	Main Mechanisms/Targets	Key Findings	Ref.
**30**–**35**	*Centaurea bruguieriana* subsp. *belangeriana* (Bornm) DC. (Asteraceae)	EtAc (Aerial)	In vitro and in silico	S-LOX inhibition	↓ LOX	[103]
**36**–**39**	*Vernoniastrum migeodii* (S. Moore) Isawumi (Asteraceae)	MeOH (Aerial)	LPS-stimulated A549 cells	Cytokine modulation	**36**–**38**: ↓ IL-6 *mRNA* expression, **39**: ↑ IL-6 and IL-8 levels	[104]
**40**	*Gymnocoronis spilanthoides* var. *spilanthoides* (Asteraceae)	DCM (Aerial)	LPS-stimulated murine macrophages	NF-κB, IRF	↓ TNF, NO; ↑ IL-10; synergistic with benznidazole	[106]
**41** and **42**	*Carpesium lipskyi* (Winkl.) C. *(Asteraceae)*	EtOH (Flower)	RAW264.7 cells	NF-κB, MAPK, Nrf2	↓ NO, iNOS, COX-2; antioxidant activation	[107]
**43**	*Achillea santolina* L. (Asteraceae)	MeOH (Flower)	In vitro and in vivo	COX-2, HO-1	↓ COX-2, IL-1β, and IL-10	[105]

Note: “↑” indicates upregulation or increase; “↓” indicates downregulation or decrease.

**Table 4:** Summary of anticancer activities of Asteraceae plant extracts highlighting their plant source, experimental models, molecular targets, and reported biological effects.

Extracts (Plant Parts Used)	Plant Species	Cancer Model/Cell Lines	Main Mechanisms/Targets	Key Findings	Ref.
MeOH (Aerial)	*Achillea multifida* (Asteraceae)	MCF-7, MDA-MB-231	DNA damage, apoptosis	Induces apoptosis and mitotic catastrophe	[130]
MeOH (Aerial)	*Vernonia leopoldii* (Sch. Blip. Ex Walp) Vatke (Asteraceae)	HepG2, MCF-7	Apoptosis, autophagy	Cell cycle arrest, reduced migration	[132]
Aqueous (Roots and Leaves)	*Jurinea dolomiaea* Boiss (Asteraceae)	EAC cells	Apoptosis	Significant growth inhibition	[133]
EtAc (Leaves)	*Vernonia amygdalina Delile* (Asteraceae)	PANC-1	PI3K/mTOR, p53, Bcl-2	Synergistic apoptosis and G1 arrest	[134]
EtOH (Roots)	*Cichorium endivia* L. (Asteraceae)	HepG2, Panc-1	Apoptosis, S-phase arrest	Strong selective cytotoxicity	[135]
MeOH (Seeds)	*Cichorium intybus* L. (Asteraceae)	MDA-MB-231	ROS, mitochondrial pathway	Enhanced apoptosis	[136]
MeOH (Aerial)	*Artemisia herba-alba* Asso. (Asteraceae)	CRC cells	PI3K/AKT/mTOR	G2/M arrest, apoptosis	[137]
EtOH, ACN, n-hex, and TCM (Aerial)	*Helichrysum sanguineum* (L.) Kostel. (Asteraceae)	MCF-7 cells	Apoptosis, cell cycle	Apoptosis and cell cycle arrest	[138]
EtOH (Leaves)	*Artemisia judaica* L. (Asteraceae)		ROS, caspase-3	Mitochondrial apoptosis	[139]
EtAc (Leaves)	*Sphaeranthus amaranthoides* Burm. f. (Asteraceae)	AGS, HT-29	Mitochondrial apoptosis	Caspase activation, G0/G1 arrest	[140]
EtOH (Aerial)	*Artemisia fragrantissima* Jacq. ex Willd. (Asteraceae)	A549	Caspase-3, Bcl-2	G2/M arrest, apoptosis	[141]
Aqueous (Aerial)	*Artemisia turcomanica* Gand. (Asteraceae)		Apoptosis, cell cycle	Growth inhibition, selective toxicity	[142]
MeOH (Leaves)	*Adenoon indicum* Dalzell (Asteraceae)	HT-29	G_2_/M, S phase	Induced cell cycle arrest, apoptosis	[158]
EtOH (Leaves and Flowers)	*Tanacetum vulgare* L. (Asteraceae)	HT-29	Genotoxic stress	Apoptosis, tumor reduction in vivo	[143]
EtOH (Leaves	*Elephantopus scaber* L. (Asteraceae)	T47D	Apoptosis, cell cycle	Inhibit ERα and the Nrf2 complex	[144]
MeOH (Aerial)	*Taraxacum officinale* (Asteraceae)	MCF-7	ROS, cell cycle (G1 phase), apoptosis	Reduced cell viability, membrane and mitochondrial permeability, cytochrome c release, and apoptosis.	[145]
Aqueous (Aerial)		A549	Apoptosis	G1/S phase cell cycle arrest and apoptosis	[149]
EtAc (Aerial)	*Echinops Shakrokii S.A. Ahmad* (Asteraceae)	MCF-7	Apoptosis, caspase-3, macrophage	Induced apoptosis and enhanced immune responses	[146]
Aqueous (Aerial)	*Achillea biebersteinii* Afan. (Asteraceae)	*DKFZ-EP1NS*	NF-κB, TRPC6	G0/G1 arrest, apoptosis	[147]
EtOH (Leaves & Stems)	*Anacyclus pyrethrum* (L.) Link. (Asteraceae)	A549	caspase-3, caspase-9, gene Bcl-2, cell cycle (G1 phase)	G1 phase cell cycle arrest, upregulate caspase-3 and caspase-9 and downregulate gene Bcl-2	[148]
MeOH (NR)	Centaurea saligna (*K.Koch*) *Wagenitz.* (*Asteraceae*),	HT-29 and A549	caspase-3, PARP, p53	Increased apoptotic indices, caspase-3 activation, cleaved PARP, and 836 elevated p53 expression	[150]
	*Centaurea bingoelensis* Behçet & İlçim. (Asteraceae)				
	*Centaurea pyrrhoblephara* (*Boiss.*) *Wagenitz* (Asteraceae)				
DCM & EtOH (NR)	*Centaurea lycaonica* Boiss. & Heldr. (Asteraceae)	HeLa	caspase-3 and -9 activity and an elevated Bax/Bcl-2	Apoptosis, increased caspase-3 and -9 activity and an elevated Bax/Bcl-2 ratio	[151]
EtOH (Aerial)	*Ageratum fastigiatum* (Gardner) R.M.King & H.Rob. (Asteraceae)	MDA-MB-231	Sub-G0/G1 cells and apoptosis	Reduced cell viability, inhibited migration, and decreased colony formation, increased sub-G0/G1 cells, apoptosis	[152]
EtOH (Leaves)	*Vernonia amygdalina* (Asteraceae)	Hs578T	Apoptosis, cell cycle (G2/M phase)	apoptosis, cell cycle progression	[153]
EtOH (Seeds)	*Artemisia absinthium* L. (Asteraceae)	HepG-2	PI3K/Akt/mTOR	cell cycle arrest, downregulated PI3K/Akt/mTOR	[154]
Aqueous (Stems)	*Gundelia tournefortii* L.	A549	BAX, HSP90	Apoptosis induction	[155]
EO (Branch & Leaves)	*Conyza bonariensis* (L.) Cronquist (Asteraceae)	SK-MEL-28	Apoptosis, ERK1/2, JNK, p38 MAPK, NF-κB, and PKB/AKT	Apoptosis, modulated ERK1/2, JNK, p38 MAPK, NF-κB, and PKB/AKT	[80]
EO (Aerial)		SK-MEL-28	ROS	ROS induction and embryotoxicity in vivo	[156]
EO (Leaves & Flowers)	*Acmella paniculata* (Wall. ex DC.) R.K. Jansen (Asteraceae)	SCC9	Apoptosis, p53	Induced apoptosis	[157]

**Table 5:** Summary of anticancer activities of investigated Asteraceae phytochemicals, highlighting their experimental models, molecular targets, and reported biological effects.

Tested Phytochemicals	Extract (Parts Used)	Plant Species	Model/System	Main Mechanisms/Targets	Key Findings	Ref.
**67**	MeOH (Aerial)	*Ainsliaea macrocephala* (Mattf.) Y.C.Tseng (Asteraceae)	HT-29 and HCT-116	Bcl-2, CDK2, cyclin A2, caspase-3 and Bax, apoptosis, PPARγ	Apoptosis and tumor reduction in vivo	[159]
**68**	EtOH (Leaves)	*Gerbera piloselloides* (L.) Cass (Asteraceae)	HT-29	Apoptosis, cell cycle, ROS, PARP, Cyclin D1	Apoptosis and tumor reduction in vivo	[160]
**69**	MeOH (Aerial)	*Artemisia dracunculus* (L.) (Asteraceae)	RT-112, HTB9, and HT1376	caspase-3, apoptosis and Erk	Apoptosis and influenced the Erk signaling pathway	[161]
**70**	MeOH (Aerial)	*Centrapalus pauciflorus* (Willd.) H. Rob. (Asteraceae).	HeLa	Apoptosis, migration inhibition	Anti-metastatic effects	[162]
**71**	EtOH (Aerial)	*Centaurothamnus maximus* (Forssk.) Wagenitz & Dittrich (Asteraceae)	A549	Cell cycle (G2/M phase), apoptosis	Induced G2/M phase cell cycle arrest and triggered apoptosis	[163]
**72**	EtOH (Whole Plant)	*Eupatorium chinense* L. (Asteraceae)	AGS	DNA-PK/AKT/p53, cell cycle (G0/G1 phase), p21, CDK4, cyclin D, apoptosis	Cell cycle arrest, increased Bax and decreased Bcl-2	[164]
**73**	MeOH (Roots)	*Inula helenium* L. (Asteraceae)		Cell cycle (G2/M phase), apoptosis, cyclin B1, CDC2, pyroptosis, PARP	Cell cycle arrest, triggered both apoptosis and pyroptosis	[165]
**74**	MeOH (Leaves)	*Himalaiella heteromalla* (D.Don) Raab-Straube (Asteraceae)	MIA 1025 PaCa-2	Cell cycle (G2/M phase), apoptosis, mitochondrial function, Bcl-2	Apoptosis induction and cell cycle arrest	[166]
**75**	TCM (Aerial)	*Inula oculus-christi* L. (Asteraceae)	HL-60	Cell cycle (G1 phase), apoptosis, ROS	Apoptosis induction and cell cycle arrest	[167]
**76**	NR	*Matricaria chamomilla* L. (Asteraceae)	HepG2	Cell cycle (G2/M phase), apoptosis, Cyclin B1 and CDK1, FasL, cleaved caspase-8, caspase-3, and PARP	Apoptosis induction and cell cycle arrest	[168]
**77**	EtOH (Aerial)	*Taraxacum officinale* (Asteraceae)	LLC, SPC-A1	Cell cycle (S phase), Bax, EMT, caspase-9, PARP1, apoptosis, Bcl-2, mitochondria, T (Treg)	Cell cycle arrest, apoptosis, anti-metastasis	[169]
**78**	NT	*Eclipta prostrata* (L.) L. (Asteraceae)	A549	Par3, PI3K/Akt/mTOR, apoptosis, cell cycle (G1 phase)	Upregulated Par3 expression and suppressed PI3K/Akt/mTOR, apoptosis	[170]
**79** and **80**	MeOH (Flowers & Leaves)	*Achillea grandifolia* Friv. (Asteraceae)	U87MG and T98G	Cell cycle (G2/M phase)	Disruption of cell cycle progression	[171]
**81**	MeOH (Seeds)	*Arctium lappa* L. (Asteraceae),	SW480, HCT15, and HCT116	Cell cycle (S-phase), GO, KEGG, CDKN1C/p57	Induced S-phase cell cycle arrest	[172]
**82**	MeOH (Aerial)	*Centaurea bruguieriana* subsp. belangeriana (Asteraceae)	U87MG, T98G	Cell cycle (G1/G0 phase),		[173]
**83**	EtAc (Aerial)	*Centaurea bruguieriana* subsp. belangeriana (DC.) Bornm. (Asteraceae)	U87 and T98	Cell cycle (S, G2/M phase),	Cell cycle arrest	[174]

## Data Availability

No new data were created or analyzed in this study.
